# *Jdp2*-deficient granule cell progenitors in the cerebellum are resistant to ROS-mediated apoptosis through xCT/Slc7a11 activation

**DOI:** 10.1038/s41598-020-61692-x

**Published:** 2020-03-18

**Authors:** Chia-Chen Ku, Kenly Wuputra, Kohsuke Kato, Wen-Hsin Lin, Jia-Bin Pan, Shih-Chieh Tsai, Che-Jung Kuo, Kan-Hung Lee, Yan-Liang Lee, Ying-Chu Lin, Shigeo Saito, Michiya Noguchi, Yukio Nakamura, Hiroyuki Miyoshi, Richard Eckner, Kyosuke Nagata, Deng-Chyang Wu, Chang-Shen Lin, Kazunari K. Yokoyama

**Affiliations:** 1 Graduate Institute of Medicine, Kaohsiung Medical University, 80708 Kaohsiung, Taiwan (R.O.C.); 2 Regenerative Medicine and Cell Therapy Research Center, Kaohsiung Medical University, 80708 Kaohsiung, Taiwan (R.O.C.); 3 Department of Infection Biology, Graduate School of Comprehensive Human Sciences, The University of Tsukuba, 305-8577 Tsukuba, Ibaraki Japan; 4 National Laboratory Animal Center, National Applied Research Laboratories (NARLabs), Xinshi Dist., 74147 Tainan, Taiwan (R.O.C.); 5 National Laboratory Animal Center, National Applied Research Laboratories (NARLabs), Nangang Dist., 11599 Taipei, Taiwan (R.O.C.); 6 Welgene Biotech., Inc., 11503 Taipei, Taiwan (R.O.C.); 7 School of Dentistry, Kaohsiung Medical University, 80708 Kaohsiung, Taiwan; 8 Saito Laboratory of Cell Technology, Yaita, 329-2192 Tochigi Japan; 9 Waseda Research Institute for Science & Engineering, Waseda University, 169-0051 Tokyo, Japan; 10 Cell Engineering Division, RIKEN BioResource Research Center, 305-0074 Tsukuba, Ibaraki Japan; 11 Department of Physiology, Keio University School of Medicine, Shinanaomachi, 168-8582 Tokyo Japan; 12 Departent of. Biochemistry & Molecular Biology, Rutgers New Jersey Medical School, The State University of New Jersey, 07-103 Newark, NJ USA; 13 Division of Gastroenterology, Department of Internal Medicine, Kaohsiung Medical University Hospital, 80708 Kaohsiung, Taiwan (R.O.C.); 14 Department of Biological Sciences, National Sun Yat-sen University, 80424 Kaohsiung, Taiwan (R.O.C.); 15 Department of Molecular Preventive Medicine, Graduate School of Medicine, The University of Tokyo, 113-8655 Tokyo, Japan; 16 Present Address: Founder of Gecoll Biomedicine Co. Ltd., Xinshi Dist., 744 Tainan, Taiwan (R.O.C.)

**Keywords:** Apoptosis, Molecular neuroscience

## Abstract

The Jun dimerization protein 2 (Jdp2) is expressed predominantly in granule cell progenitors (GCPs) in the cerebellum, as was shown in *Jdp2*-promoter-Cre transgenic mice. Cerebellum of *Jdp2*-knockout (KO) mice contains lower number of Atoh-1 positive GCPs than WT. Primary cultures of GCPs from *Jdp2*-KO mice at postnatal day 5 were more resistant to apoptosis than GCPs from wild-type mice. In *Jdp2*-KO GCPs, the levels of both the glutamate‒cystine exchanger Sc7a11 and glutathione were increased; by contrast, the activity of reactive oxygen species (ROS) was decreased; these changes confer resistance to ROS-mediated apoptosis. In the absence of Jdp2, a complex of the cyclin-dependent kinase inhibitor 1 (p21^Cip1^) and Nrf2 bound to antioxidant response elements of the Slc7a11 promoter and provide redox control to block ROS-mediated apoptosis. These findings suggest that an interplay between Jdp2, Nrf2, and p21^Cip1^ regulates the GCP apoptosis, which is one of critical events for normal development of the cerebellum.

## Introduction

Reactive oxygen species (ROS) are formed mainly through the reduction of oxygen in mitochondria. ROS include oxygen radicals and some non-radical oxygen derivatives such as hydrogen peroxide (H_2_O_2_), O3, and singlet oxygen^1,2^. ROS are also implicated in the progression of neurological diseases, including Alzheimer’s and Parkinson’s diseases, and stroke-related dementia^3–5^. The cerebellum is the most vulnerable part of the brain to developmental abnormalities after exposure to oxidants^6,7^. In the cerebellum, ROS exert a wide variety of actions, which range from involvement in cell proliferation to cell death, according to the “determination rule” of cell fate^1^. Understanding these actions of ROS during neuron development may help provide new information about the pathogenesis of neurological diseases. Intracellular ROS activity depends on the equilibrium between ROS generation and the activities of the antioxidant systems. Intracellular glutathione (GSH) scavenges a variety of ROS and is an obligate substrate of glutathione peroxidase for detoxification of H_2_O_2_^8^. The ratio of reduced GSH to oxidized glutathione disulfide (GSSG) is an important determinant of the intracellular redox state and redox signalling^9^. The biosynthesis of GSH is regulated by several genes including those encoding members of the cystine–glutamate antiporter (Xc–) system, which includes solute carrier family 7, member 11 (Slc7a11)^10–12^ as the transporter subunit and solute carrier family 3, member 2 (Slc3a2 or CD98 or 4F2) as the binding partner^13,14^. The Xc– system is a sodium-independent antiporter of cystine and glutamate that traps extracellular cystine in exchange for intracellular glutamate at a 1:1 molar ratio^15^. CD44v^16^ has been reported to be a positive regulator of Slc7a11 by recruiting OTUB1, an ovarian tumour family deubiquitinase^17^, which decreases the sensitivity of cancer cells to oxidative stress and ferroptosis^18^. Intracellular cystine is taken up by the Xc–system and reduced to cysteine, which is consumed during protein synthesis and finally to produce GSH, an important factor in redox balance^10^.

The nuclear factor erythroid 2-related factor 2 (Nrf2), which is a transcription factor in the basic leucine zipper family, controls redox homeostasis and facilitates neuronal adaptation to hostile oxidative environments^19^. Under basal conditions, Nrf2 is sequestered in the cytoplasm by the Kelch-like ECH-associated protein 1 (Keap-1). Upon stimulation by oxidants, Nrf2 is released from Keap-1 and binds to antioxidant response elements (AREs) in the nucleus, where it upregulates NAD(P)H quinone dehydrogenase 1 (NQO-1) and the genes involved in GSH production^19–21^. Slc7a11 is induced by Nrf2 in human cancers. Slc7a11 facilitates oncogenic RAS transformation by preserving the intracellular redox balance^22^. By contrast, inhibition of Slc7a11 leads to intracellular cysteine depletion and increases ROS levels, which activate non-apoptotic forms of cell death such as ferroptosis^18^. The Nrf2 complex has been recently shown to contain ADP ribosylation factor Arf as a binding partner both *in vitro* and *in vivo*^23,24^.

Nrf2 deficiency makes neurons susceptible to oxidant-induced injury^25,26^. Nrf2–ARE activity is lower in forebrain neurons than in astrocytes, and this difference is required for neuronal development^27,28^. The phase II ligand tert-butyl hydroquinone induces the expression of the ARE-containing genes Gst1 and Nqo1 in astrocytes, but not in cortical neurons. However, astrocytes can exert neuroprotective effects in a cell-nonautonomous manner^21,29^. Nrf2-regulated GSH also plays a major antioxidative role in the nervous system^30^, where it antagonizes oxidation-mediated stress and apoptosis elicited by ethanol exposure in cerebral cortical neurons^31^.

GSH levels may fluctuate during brain development. A sudden increase in GSH expression in the mouse brain occurs at postnatal day 12 (P12), after which GSH remains at a high level until adulthood^32^. By contrast, another study reported that the GSH level increases transiently during the first postnatal week, after which it returns to the basal level and then remains low during the subsequent stages of cerebellar development^33^. Therefore, the level of GSH during cerebellar development is still debated.

During embryonic and postnatal development, cerebellar subtypes seem to be generated in a sequential manner from two distinct germinal centres such as the ventricular zone (VZ) and the rhombic lip (RL)^34^. The VZ is defined by the specific expression of transcription factors, such as Mash1, neurogenins, and Ptf1A^35^. The RL is characterized by the expression of Atoh1 and Pax6^36^. During postnatal development, the VZ delaminates to bring about the secondary germinal centre, the prospective white matter^37^. By contrast, the RL progenitor migrates tangentially above the subapical surface to give rise to the external granular layer^38^. The VZ progenitors also generate all GABAergic neurons and cerebellar glial cells, whereas the RL progenitors produce all glutamatergic neurons^39^.

The Jun dimerization protein 2 (Jdp2) is a member of the activator protein 1 (AP-1)–activating transcription factor (ATF) family. Jdp2 is involved in transcriptional repression via multiple mechanisms, including DNA binding and competition with other AP-1–ATF family members, inhibition of heterodimer formation with other transcription factors, recruitment of histone deacetylase-3 (HDAC3), and inhibition of histone acetylation and histone chaperone^40–42^. Jdp2 is also a cofactor of the Nrf2‒MafK complex, which is involved in the regulation of AREs and ROS production in mouse embryonic fibroblasts (MEFs)^43^. Jdp2 binds directly to the core sequence of AREs, where it associates with Nrf2 and MafK. Binding of Jdp2 to AREs promotes the transcription of genes encoding antioxidant and detoxification enzymes^43^, through which Jdp2 suppresses ROS production and confers a more reduced intracellular environment in MEFs.

The proper type, location, and number of neurons are determined by the critical interplay between transcription factors and signalling, which ensures the adequate development of the cerebellum^37,38,44^. However, the role of Jdp2 in the development and cell death of cerebellar neurons remains largely unknown. In the study reported here, we found that the *Jdp2* promoter is activated predominantly in the mouse cerebellum. *Jdp2* deficiency caused less apoptosis via the dysregulation of antioxidation activity in granule cell progenitors (GCPs) from the cerebellum in knockout (KO) mice than in those from wild-type (WT) mice. These effects were associated with abnormal cerebellar development in the *Jdp2*-KO mice.

## Results

### Predominant activation of the Jdp2 promoter in the cerebellum

**Figure 1:**
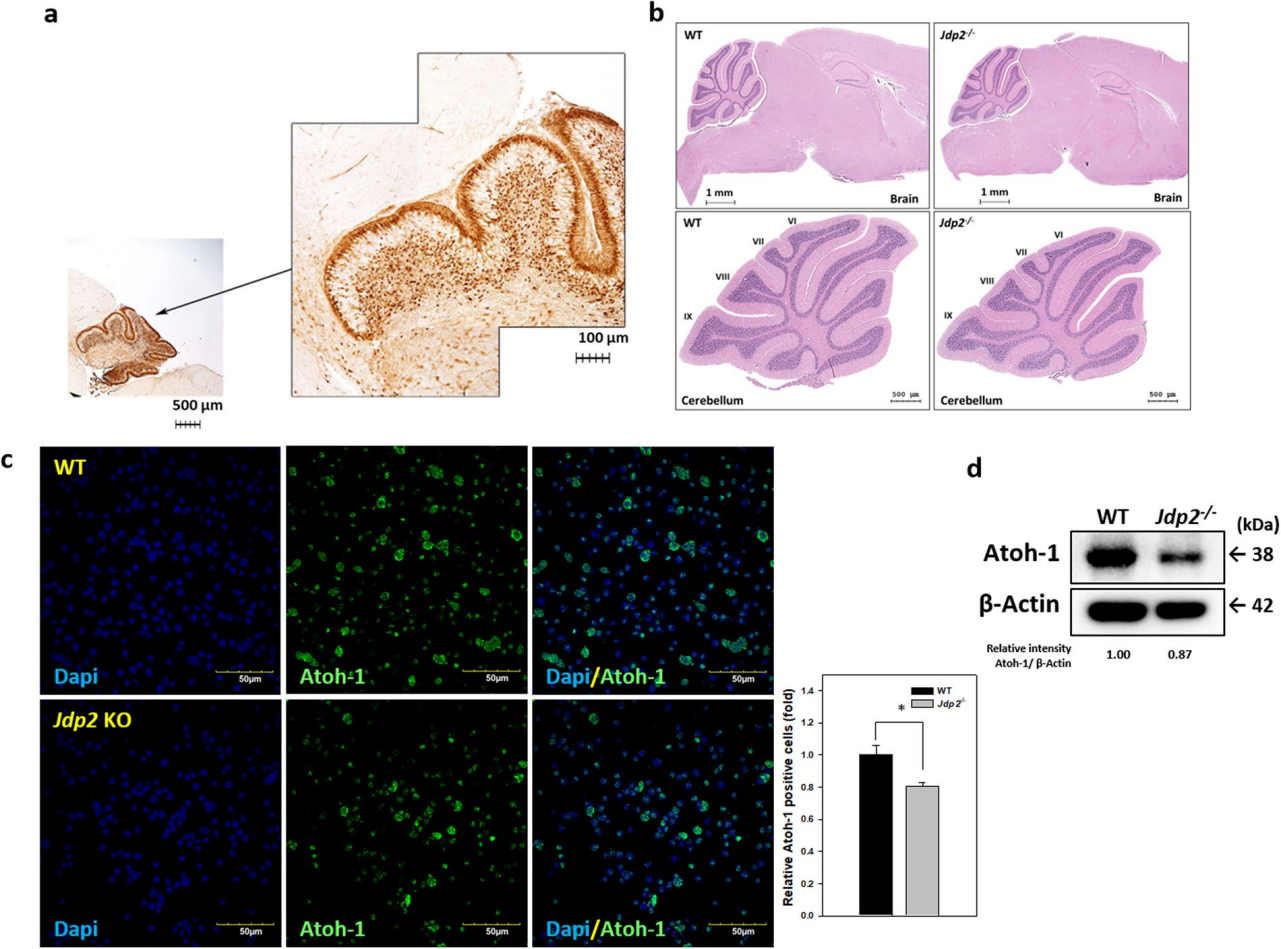
No loss or surplus of cerebellar lobes was observed in granule cells from *Jdp2*-KO mice, where lower expression of Atoh-1 was detected in these cells. (**a**) β-Gal staining at P6 in Jdp2-Cre/ROS26R mice showed signals in the regions rich in cerebellar granule cells (magnification 20×) (GCPs; right panel; magnification 100×). (**b**) Haematoxylin and eosin staining of the cerebellum from WT 129-C57/BL6J and *Jdp2*-KO 129-C57/BL6J mice. (**c**) GCPs were prepared from WT and *Jdp2*-KO mice and stained for Atoh-1. Atoh-1-stained cells were quantified according to the calculation presented in Materials and Methods. *p < 0.05. (**d**) Western blot analysis showed that the expression of Atoh-1 was about 87% lower in GCPs from *Jdp2*-KO mice than in those from WT mice.

*Jdp2* promoter-Cre transgenic mice were obtained and crossed with ROSA26R^45^ and Z/EG reporter mice^46^. Sibling breeding of double-transgenic mice of the *Jpd2* promoter-Cre/ZEG or *Jdp2* promoter-Cre/ROSA26R strains was confirmed by LacZ (β-Gal) staining, green fluorescent protein (GFP) signal detection, and polymerase chain reaction (PCR) genotyping. Previously we reported that *Jdp2* promoter-Cre/ZEG mice expressed GFP signals in the brain at postnatal day 24 (P24), predominantly in the cerebellum^47^. The β-Gal staining showed that signals are also present in the cerebellum of *Jdp2* promoter-Cre/ROSA26R mice at P6 (Fig. 1a). The expression of *Jdp2*-specific transcripts was also detected in the brain, as reported previously^48^, which confirms that the β-Gal or GFP activities observed in the cerebellum are derived from the *Jdp2* promoter. These data suggest that the *Jdp2* promoter is activated in the cerebellum, particularly in the region rich in granule cells.

### No loss or surplus of lobes in the cerebellum of Jdp2-KO mice

Next, we compared the cerebellar morphology between WT and *Jdp2*-KO mice^49^. There was no significant loss or surplus of lobes between WT and *Jdp2*-KO cerebella (Fig. 1b). In the early development of the cerebellum, granule cell progenitors (GCPs) with Atoh-1 expression represent >80% of the cell population of the external germinal layer^49^. Immunofluorescent staining and western blot demonstrated that both the Atoh-1 positive cells (Fig. 1c) and Atoh-1 expression level (Fig. 1d) were decreased in *Jdp2*-KO GCPs as compared with those in WT primary GCPs. Furthermore, the results of RNA sequencing and RT-PCR showed that the expression of some cerebellar (e.g., *Zic1, Zic2, Ntrk3, NGF*, and *BDNF*) and neuronal (e.g., *Gabra1, Gabra 2*, and *Glra2*) genes were deregulated (Supplementary Fig. 1a,b). These observations suggest that Jdp2 plays a role in the maturation and differentiation of GCPs, which is critical for lobe development in the cerebellum.

### GCP resistance to apoptosis via Jdp2 deficiency

**Figure 2:**
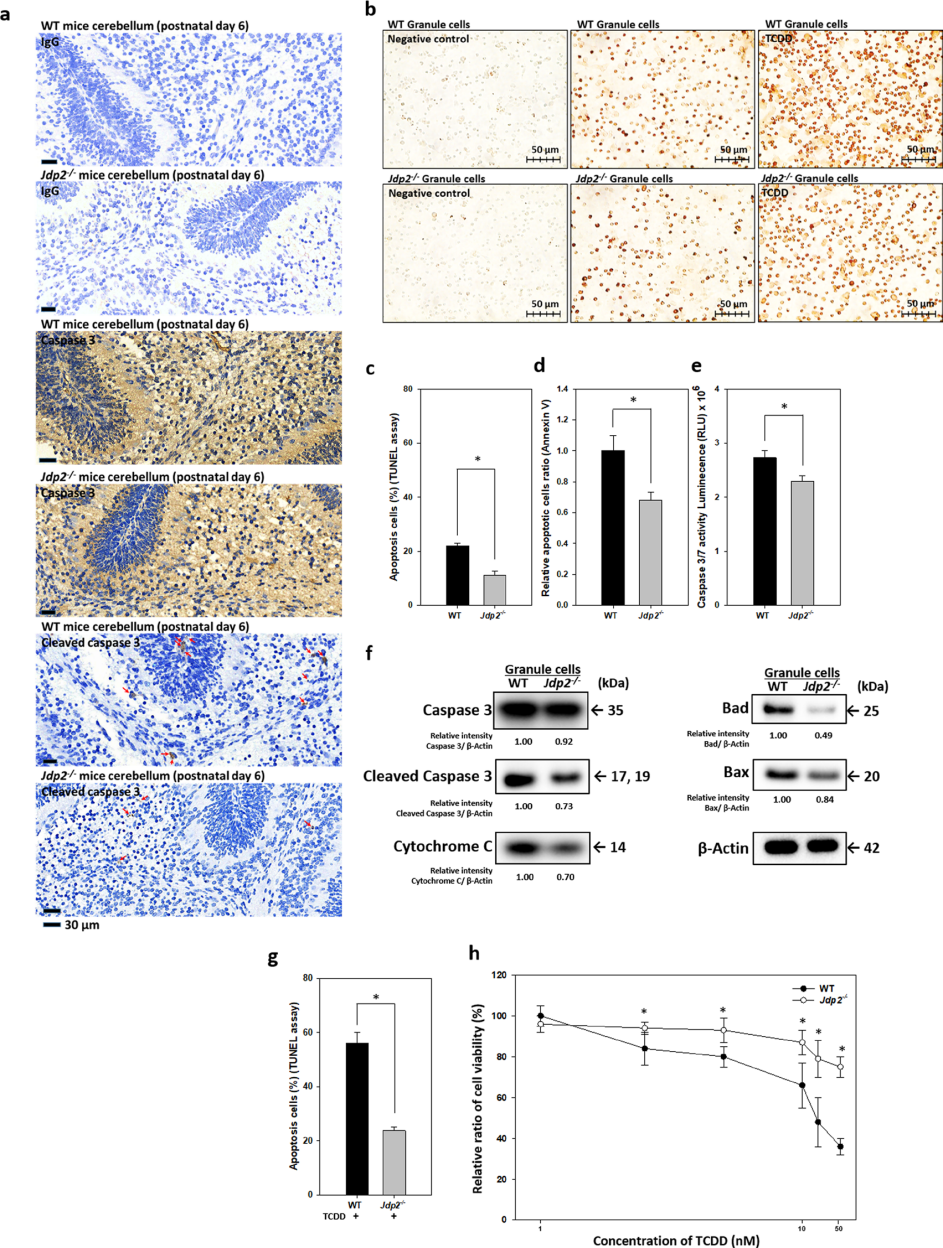
Decreased apoptosis in *Jdp2*-KO GCPs. (**a**) Cerebellum sections from WT and *Jdp2*-KO mice were stained with anti-caspase 3 and anti-cleaved caspase 3 antibodies and with control IgG, as described in Materials and Methods. The red arrows indicate the granule cells stained by the anti-cleaved caspase 3 antibody. (**b**) GCPs from WT and *Jdp2*-KO mice were incubated with or without TCDD (10 nM) for 24 h, and the cells were stained for TUNEL assays as described in Materials and Methods. (**c**) Quantification of apoptotic cells from the images of panel (**b**). Each value represents the mean ± SEM (n = 3); *p < 0.05. (**d**) Annexin V staining of WT and *Jdp2*-KO GCPs was performed as described in Materials and Methods. Each value represents the mean ± SEM (n = 3); *p < 0.05. (**e**) Caspase 3/7 activities were measured in 3 × 10^5^ WT and *Jdp2*-KO GCPs in 96-well plates. Each value represents the mean ± SEM (n = 3); *p < 0.05. (**f**) Comparative expression of apoptosis-related proteins in WT and *Jdp2*-KO GCPs (n = 5). The right lane indicates the apparent molecular weight. The relative intensity of each band was calculated relative to β-actin. (**g**) Quantification of apoptotic cells in TCDD (50 nM)-treated WT and *Jdp2*-KO GCPs. Each value represents the mean ± SEM (n = 3); *p < 0.05. (**h**) Cell viability of WT and *Jdp2*-KO GCPs with increasing doses of TCDD (1.0‒50 nM). Each value represents the mean ± SEM (n = 3); *p < 0.05.

To examine the role of Jdp2 in GCP homeostasis, which is important for normal cerebellum development, the apoptosis of GCPs was examined. The immunostaining with anti-activated Caspase 3 antibody showed that the cerebellum section from WT mice exhibited the higher percentage of stained granule cells than those from *Jdp2*-KO mice at postnatal day 6 (Fig. 2a). The data pertaining to the staining using antibodies against caspase 3 and cleaved caspase 3 also showed a 1.5‒2-fold percentage of granule cells with staining in WT cerebella compared with the *Jdp2*-KO counterparts. However, the cells that were stained with isotypic control IgG showed almost no staining. To confirm this result *in vivo*, TUNEL assay also showed that the percentage of apoptotic cells in WT and *Jdp2*-KO GCPs was 22% and 11%, respectively (Fig. 2b,c). Annexin V assays confirmed a decrease in apoptosis in *Jdp2*-KO GCPs (Fig. 2d). Compared with WT GCPs, *Jdp2*-KO GCPs showed a 20% reduction in the activities of caspase 3 and 7 (Fig. 2e) and decreased protein levels of caspase 3, cleaved caspase 3, Bad, Bax, and cytochrome C (Fig. 2f). The co-staining with anti-Atoh1 (Alexa-488) and anti-cleaved caspase 3 (Alexa-594) were also detected (data not shown). These findings suggest that *Jdp2*-KO GCPs undergo less apoptosis than do WT GCPs *in vivo* and *in vitro*.

It is reported that TCDD exposure to the developing brain causes cognitive disability and motor dysfunction^50^. Here, we found that TCDD treatment (50 nM) increased the number of apoptotic cells to a greater extent in WT (57%) vs. *Jdp2*-KO (23%) GCPs (Fig. 2g). A trypan blue dye-exclusion cell viability assay also showed that *Jdp2*-KO GCPs were more resistant to TCDD treatment (1.0–50 nM) than were WT GCPs (Fig. 2h). These results indicate that *Jdp2*-KO GCPs have higher capacity in tolerate TCDD-induced stress when compared with WT GCPs.

### Increased antioxidation activity in Jdp2-KO GCPs

**Figure 3:**
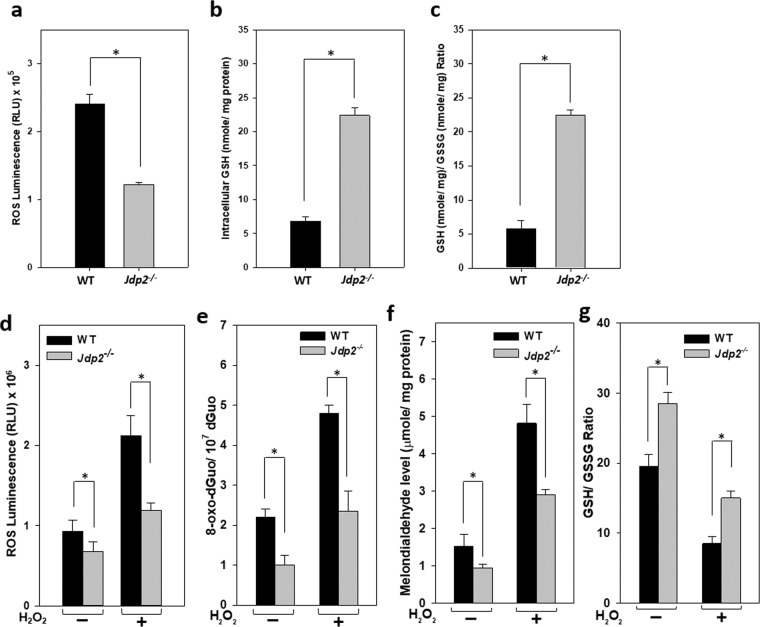
Reduced oxidative stress in *Jdp*2-KO GCPs. (**a**) ROS levels in WT and *Jdp*2-KO GCPs were measured using the ROS-Glo H_2_O_2_ assay described in Materials and Methods. The intracellular GSH level (**b**) and GSH/GSSG ratio (**c**) were measured and calculated as described in Materials and Methods. (**d**) ROS levels in WT and Jdp2-KO GCPs in the presence or absence of H_2_O_2_ (100 μM) for 2 h before harvesting cells for 24 h. (**e**) Levels of 8-oxo-dGuo in WT and *Jdp2-*KO GCPs in the presence or absence of H_2_O_2_ (100 μM) for 2 h before harvesting cells for 24 h. (**f**) MDA levels in the presence or absence of H_2_O_2_ (100 µM) for 2 h before the cultivation of cells for 24 h. (**g**) GSH/GSSG ratio in the presence or absence of H_2_O_2_ (100 μM) for 2 h before the cultivation of cells for 24 h. Data are presented as the mean ± SEM (n = 3); *p < 0.05.

It is known that TCDD induces neurotoxicity through induction of oxidative stress^51–53^. Therefore, we examined the levels of ROS and GSH/GSSG in WT and *Jdp2*-KO GCPs. We found that ROS level was 50% lower in *Jdp2*-KO GCPs than it was in WT GCPs (Fig. 3a). The levels of TCDD-induced ROS were also lower in *Jdp2*-KO than they were in WT GCPs (Supplementary Fig. 3a). By contrast, both the GSH level (Fig. 3b, Supplementary Fig. 2b) and GSH/GSSG ratio (Fig. 3c) were higher in *Jdp2*-KO GCPs than WT GCPs, which might contribute to the reduced oxidative stress, in *Jdp2*-KO GCPs.

To confirm this notion, H_2_O_2_-induced oxidative responses, such as the induction of ROS and DNA/lipid oxidation and the consumption of GSH, were compared between WT and *Jdp2*-KO GCPs. In the presence of 100 μM H_2_O_2_, both the basal and H_2_O_2_-induced ROS activities were lower in *Jdp2*-KO than they were in WT GCPs (Fig. 3d). Moreover, both the basal and H_2_O_2_-induced 8-oxo-dGuo (an indicator of DNA oxidation; Fig. 3e) and MDA (an indicator of lipid peroxidation; Fig. 3f) levels were consistently lower in *Jdp2*-KO GCPs than in WT GCPs. By contrast, the ratio of GSH/GSSG was still higher in *Jdp2*-KO than it was in WT GCPs after H_2_O_2_ treatment (Fig. 3g). However, the possibility that the N-acetyl cysteine in the culture medium to avoid the cell damages could enhance the antioxidation response and increase the level of GSH is also possible. Thus, we measured the comparative expression of antioxidation-related transcription factors and enzymes in the presence or absence of NAC. We found that this influence by NAC is not significant and then our condition is better to obtain the better cell survival (Supplementary Fig. 3).

**Figure 4:**
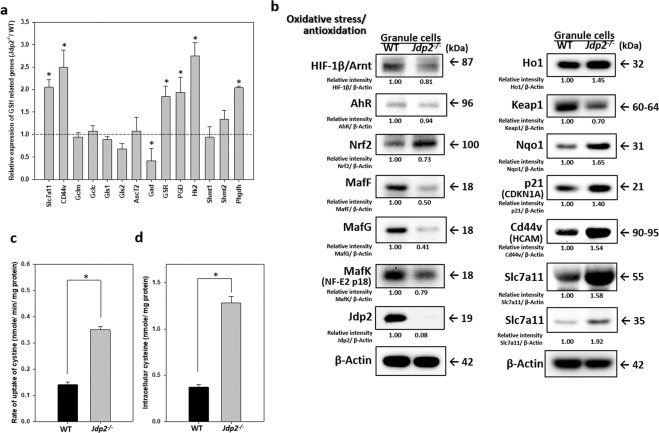
Activation of GSH anabolism genes with increased cystine uptake and intracellular cysteine level in *Jdp2*-KO GCPs. (**a**) Comparative mRNA expression of GSH production-related genes. Gene expression in WT GCPs was set as 1.0. Gsr; glutathione reductase, Pgd; 6-phosphoglycero dehydrogenase, Hk2; hexokinase 2, Phgdh; phosphoglycerate dehydrogenase, Gclm; glutamate–cysteine ligase complex modifier subunit, Gclc; glutamate–cysteine ligase complex catalytic subunit, G6p; glucose-6-phosphate, Gpx4; glutathione peroxidase 4; 3-Pg; 3-phosphoglycerol; GSH; glutathione-SH. Each value represents the mean ± SEM (n = 3); *p < 0.05. (**b**) Representative protein expression in the pathways for oxidative stress and antioxidative responses. Aliquots of WT and *Jdp2*-KO GCPs proteins were examined by western blotting (n = 3; n means three independent samples) after cultivation of these cells for 24 h. The right lane indicates the apparent molecular weight. The relative intensity was calculated relative to β-actin. Cystine uptake (**c**) and intracellular content of cysteine (**d**) in WT and *Jdp*2-KO GCPs proteins were measured as described in Materials and Methods. Each value represents the mean ± SEM (n = 3); *p < 0.05.

To clarify the mechanism underlying the increased in GSH level in *Jdp2*-KO GCPs, we examined the expression of the genes involved in GSH biogenesis (Supplementary Fig. 4)^8,9^. Both RT–PCR and western blot analyses showed that the expression of Slc7a11 and Cd44*v* was increased in *Jdp2*-KO GCPs (Fig. 4a,b), which were consistent with the elevation of cystine uptake rate (Fig. 4c) and intracellular cysteine level (Fig. 4d) in *Jdp2*-KO GCPs. Moreover, the mRNAs of glutathione reductase (Gsr), 6-phosphogluconate dehydrogenase (Pgd), hexokinase 2 (Hk2), and phosphoglycerate dehydrogenase (Phgdh) were upregulated in *Jdp2*-KO GCPs, this might provide additional NADPH and serine/glycine for GSH anabolism (Supplementary Fig. 4 and Fig. 4a). The levels of expression of Nrf2, Ho1, and Nqo1 proteins were increased by more than twofold, whereas the expression of small Maf proteins (G, F, and K) and Keap-1 were reduced in *Jdp2*-KO GCPs (Fig. 4b). These results suggest that *Jdp2*-KO GCPs exhibit an enhanced antioxidative activity.

### Differential regulation of *Slc7a11* promoter and ARE activity by Jdp2

**Figure 5:**
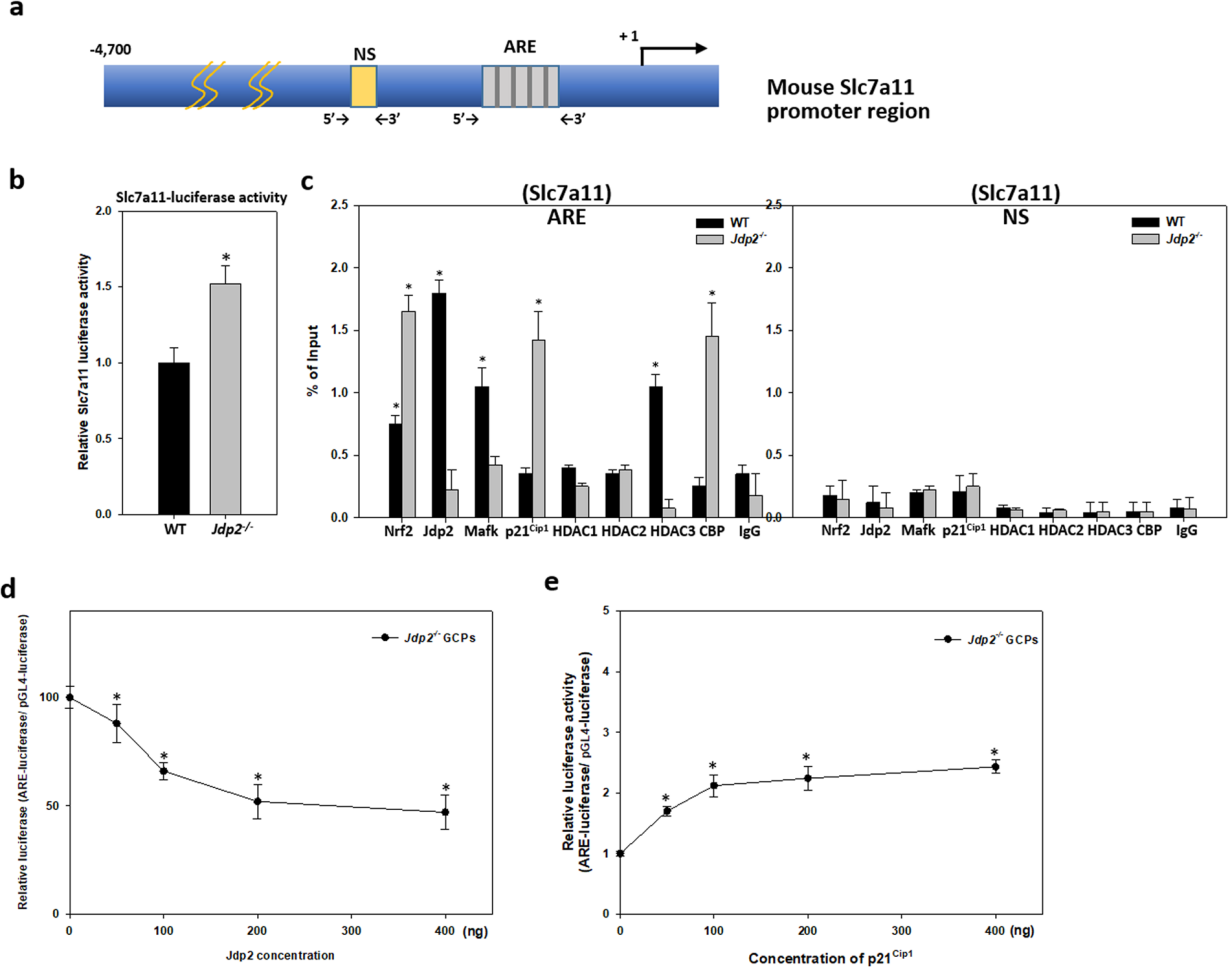
Differential regulation of *Slc7a11* promoter activity in WT and *Jdp2*-KO GCPs by Jdp2, Nrf2, and p21^Cip1^ through the ARE. (**a**) Schematic representation of the mouse *Slc7a11* promoter and the positions of qPCR primers for the ChIP assay. ARE, antioxidative responsive element, NS, non-specific sequence motifs (as a negative control). (**b**) *Slc7a11* promoter–luciferase activity (pGL3-4.7); mouse xCT–luciferase (ref. ^10^) was elevated in *Jdp2*-KO GCPs. Each value represents the mean ± SEM (n = 3); *p < 0.05. (**c**) ChIP–qPCR analyses were performed on chromatin extracts from WT and *Jdp2*-KO GCPs using the indicated antibodies and normal IgG (as a negative control) as described in Materials and Methods. Each value represents the mean ± SEM (n = 5); *p < 0.05. (**d**) and (**e**) Effects of Jdp2 and p21Cip1 on ARE–luciferase activity. pGL-hQR25-luciferase (100 ng) plus 0–400 ng of pcDNA-Jdp2 (**d**) or pcDNA-p21^Cip1^ (**e**) were transfected into Jdp2-KO GCPs. One day after transfection, cells were collected, and luciferase activity was measured as described in Materials and Methods. Values represent the mean ± SEM. (n = 3). *p < 0.05.

To investigate the role of Jdp2 on the expression of *Slc7a11* gene, which contains an ARE on the promoter region (Fig. 5a), *pGL3-4.7* (*Slc7a11* promoter-luciferase reporter plasmid) was transfected into WT and Jdp2-KO GCPs. The results showed that *Slac7a11* promoter activity was higher in *Jdp2*-KO GCPs (Fig. 5b), suggesting that Jdp2 might negatively regulate Slc7a11 promoter activity. Previously, we reported that Jdp2 interacts physically with Nrf2 and MafK to regulate the expression of *Ho1* and *Nqo1* through AREs in MEFs^43^. The p21^Cip1^ can also interact with Nrf2 to increase antioxidative activity^54^. Therefore, chromatin immunoprecipitation (ChIP)–qPCR experiments were conducted to investigate the recruitment of Nrf2, MafK, p21^Cip1^, and Jdp2 to the ARE site of *Slc7a11* promoter. We found that the binding of Nrf2 and p21^Cip1^ was increased in *Jdp2*-KO GCPs (Fig. 5c, left panel). Moreover, the recruitment of the histone acetyltransferase CBP was also increased. By contrast, the binding of Jdp2, MafK, and of the histone deacetylase HDAC3 was enhanced in WT GCPs (Fig. 5c, left panel). The recruitment of these factors to a nonspecific sequence of *Slc7a11* promoter was not observed or was indistinguishable between the WT and *Jdp2*-KO GCPs (Fig. 5c, right panel). Furthermore, reconstituting Jdp2 expression in *Jdp2*-KO GCPs suppressed ARE activity in a dose-dependent manner (Fig. 5d) and overexpression of p21^Cip1^ increased ARE activity (Fig. 5e). These results indicate that Nrf2 and p21^Cip1^ positively but Jdp2 negatively regulate *Slc7a11* promoter activity through ARE. Protein expressions of Nrf2, p21^Cip1^, Cd44v and Slc7a11 were also increased in *Jdp2*-KO GCPs, compared with those of WT GCPs (Fig. 4b)

### Essential role of p21^Cip1^ in reducing ROS generation and apoptosis in GCPs

**Figure 6:**
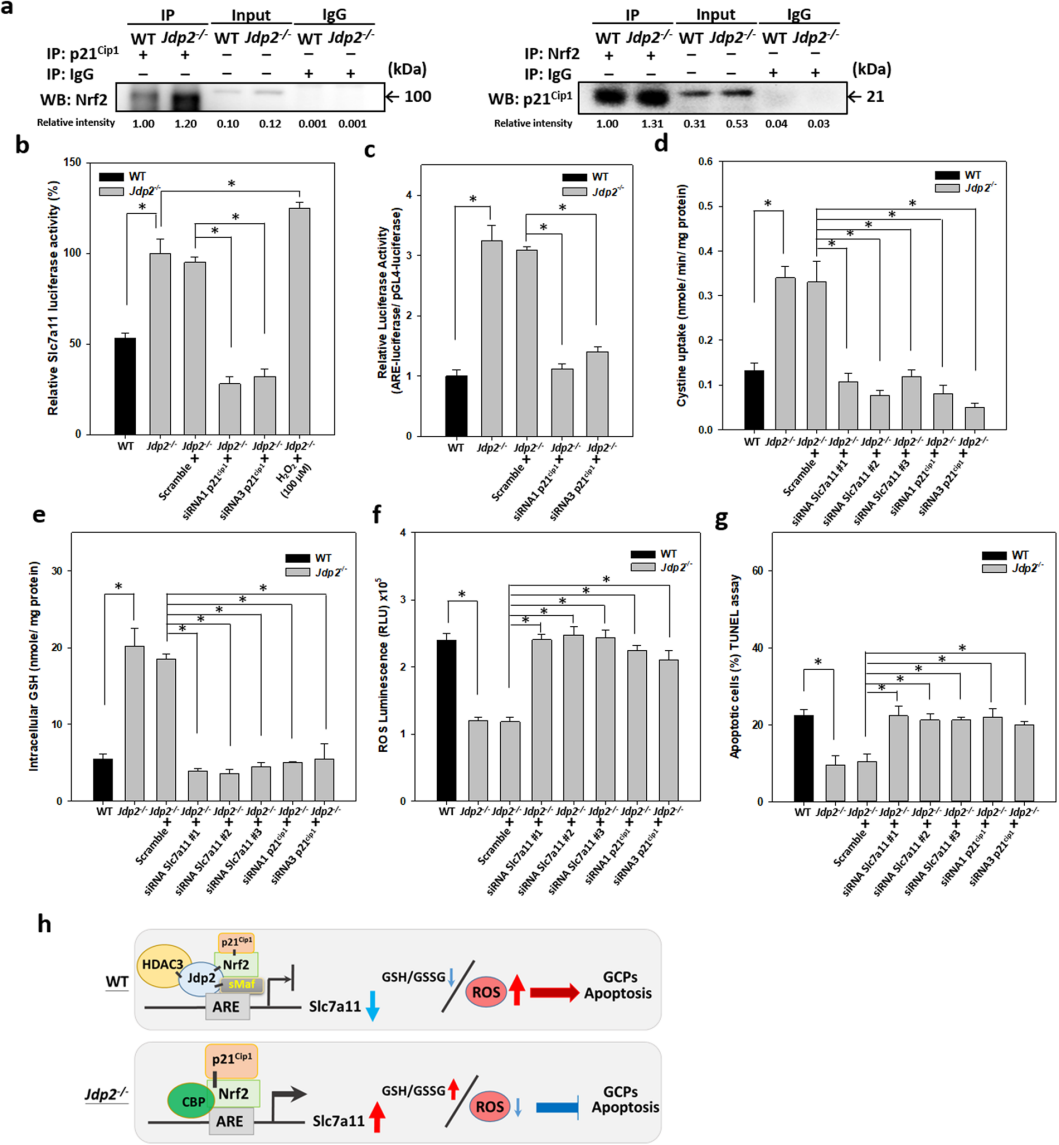
Role of p21^Cip1^ in the antioxidative response, which decreases ROS generation and apoptosis in *Jdp2-* KO GCPs. (**a**) Increased protein interaction between endogenous p21^Cip1^ and Nrf2 in *Jdp2*-KO GCPs. Cell lysates (120 μg) from WT and *Jdp2*-KO GCPs were immunoprecipitated with anti-p21^Cip1^ (left panel) or anti-Nrf2 (right panel) antibodies, and the bound proteins were blotted with anti-Nrf2 (left panel) or anti-p21^Cip1^ (right panel) antibodies as described in Materials and Methods. IgG was used as a negative control. The relative intensity was calculated relative to β-actin. (**b**–**g**) Effects of siRNA against p21^Cip1^on the *Slc7a11* promoter (**b**) and ARE (**c**) activities, cystine uptake (**d**), intracellular level of GSH (**e**), ROS production (**f**) and apoptosis (**g**). WT and *Jdp2*-KO GCPs were transfected with 30 pmol of p21^Cip1^ siRNA (siRNA 1 or 3), Slc7a11 siRNA (#1, #2, and #3), or control scrambled siRNA for 24 h, and the cells were harvested and analyzed as described in Materials and Methods. Values represent the mean ± SEM (n = 3). *p < 0.05. (**h**) A schematic representation of the interactions between Jdp2, sMaf, Nrf2, and p21^Cip1^ on the ARE of the *Scl7a11* promoter in WT and *Jdp2*-KO GCPs, which regulates ROS-induced apoptosis of GCPs. In the absence of Jdp2, the endogenous interactions between Nrf2 and p21^Cip1^ at the ARE of *Slc7a11* promoter increased, which increased cystine uptake, intracellular cysteine level, and the GSH/GSSG ratio and led to decreases in ROS and apoptosis in GCPs.

Because Nrf2-p21^Cip1^ physical interaction can enhance Nrf2-regulated ARE activity^54^, therefore, we performed reciprocal immunoprecipitation and western blotting experiments to examine the interaction between Nrf2 and p21^Cip1^ in GCPs. The results showed that the binding between Nrf2 and p21^Cip1^ was enhanced in *Jdp2*-KO GCPs (Fig. 6a). The role of the Nrf2–p21^Cip1^ complex in the enhanced antioxidation capacity of *Jdp2*-KO GCPs was validated by knockdown experiments. p21^Cip1^-specific, but not scrambled, siRNAs reduced the expression of p21^Cip1^ (Supplementary Fig. 5a), the activities of ARE (Fig. 6c) and *Slc7a11* promoter (Fig. 6b), cystine uptake (Fig. 6d), and GSH levels (Fig. 6e). The expression of *Slc7a11* mRNA was also downregulated by p21^Cip1^ siRNA (Supplementary Fig. 5b). As a result, the levels of ROS (Fig. 6f) and apoptosis (Fig. 6g) were increased by p21^Cip1^ knockdown in *Jdp2*-KO GCPs.

We also used siRNA against the *xCT/Slc7a11* transporter gene to repress its expression. Treatment with siRNAs *Slc7a11* #1, #2, and #3 reduced the expression of Slc7a11 protein, as examined using the three different antibodies, ARG 57998, bs-6883R, and CST #98051. By contrast, the scrambled siRNA did not reduce the expression of Slc7a11 protein (Supplementary Figure 5c). All three siRNAs against Slc7a11 reduced the uptake of cystine and intracellular GSH levels (Fig. 6d,e) and increased ROS production and apoptosis (Fig. 6f,g) in *Jdp2*-KO GCPs. These findings suggest that p21^Cip1^ and Slc7a11 play a key role in protecting GCPs from ROS-induced apoptosis in the absence of Jdp2.

## Discussion

Our results showed the different functions of Jdp2 in GCPs in WT and *Jdp2*-KO mice as the *Jdp2*-promoter-Cre mice expressed GFP and LacZ in GCPs (Fig. 1). The primary GCPs from *Jdp2*-KO mice exhibited a weaker staining for Atoh-1 than did WT GCPs (GCPs; Fig. 1c,d,). The levels of some cerebellum-specific markers, such as the zinc finger protein 1 (*Zic*1)^55^ and *Zic2*^55^, were lower in *Jdp2-*KO GCPs (Supplementary Fig. 1), suggesting that Jdp2 might be required to control these GCP-specific genes transcriptionally. Because both Zic1 and Zic2 are required for the development of the cerebellum^55^, their downregulation in *Jdp2*-KO GCPs might be consistent with a cell proliferation that may result in the decrease of Atoh-1-positive GCPs during the process of development (Fig. 1a‒d). In contrast, the levels of genes encoding neural factors, such as the neurotrophic receptor tyrosine kinase 3 (*Ntrk3*), the nerve growth factor (*Ngf*), the brain-derived neurotrophic factor (*Bdnf*), the GABA type A receptor alpha 1 subunit (*Gabra1*), the GABA type A receptor beta 2 subunit (*Gabrb2*), and the glycine receptor alpha 2 (*Glra2*) genes, were higher in *Jdp2*-KO GCPs compared with WT GCPs (Supplementary Fig. 1). Together, these findings suggest that Jdp2 is a cerebellar granule cell marker that blocks the neural differentiation of GCPs at the P5‒P6 stage. Here, we showed that Jdp2 contributes to programmed cell death and is crucial for proper cerebellar development (Fig. 6f–h)^56^.

In the absence of Jdp2, p21^Cip1^ enhances ARE activity in a dose-dependent manner coupled to Nrf2, to increase the anti-oxidation response (Fig. 5e). In contrast, the Nrf2–p21^Cip1^ axis-mediated ARE activity is suppressed in the presence of Jdp2 (Figs. 5d and 6c). The negative role of Jdp2 in ARE activation is illustrated by the decreased promoter activity and expression of *Slc7a11* observed in WT GCPs (Figs. 4b and 6b), which is consistent with the increased recruitment of HDAC3 to the ARE of the *Slc7a11* promoter (Fig. 5c). In contrast, CBP is recruited to the ARE in the absence of Jdp2 (Fig. 5c), which might contribute to promoter activation and expression of *Slc7a11* in *Jdp2*-KO GCPs (Figs. 4b and 5c). In the absence of Jdp2, the elevated expression of the Slc7a11-CD44v transporter could lead to increased cystine uptake, intracellular cysteine, and GSH accumulation (Fig. 3b,c; Fig. 4c,d). Our results indicate that Jdp2 is a repressor of ARE activity in GCPs, that is distinct from that of MEFs. In MEFs, Jdp2 interacts with the Nrf2–MafK complex to upregulate the ARE response, as reported previously^43^.

The Slc7a11 protein appears at two molecular weights, 55 kDa and 35 kDa, although no studies have reported any relationship between the two forms. Given this difference in molecular weight, we used three different commercially available antibodies in our study. The Sc7a11 protein is ubiquitinated *in vitro*, and this form migrates to the positions with apparent molecular weights of 55–60 kDa and >80 kDa in the human 293 cell line^17^. Therefore, we used an immunoprecipitation–western blot (IP‒WB) method to examine whether the upper 55 kDa form is the ubiquitinated protein. We found that the two antibodies ARG 57998 and bs-6883R, precipitated the 55 kDa protein, and that the CST #98051 antibody precipitated only the 35 kDa protein band from mouse GCPs (Supplementary Figs. 6a and 7).

In an attempt to characterize both proteins, the immunoprecipitated 55 kDa protein was first trapped by the MBS04751086 antibody. We then found that the 35 kDa protein, which was trapped by CST#98051, was included in the immunoprecipitates of anti-55 kDa ARG 57998. The antibodies against the 55 kDa protein precipitated the 35 kDa protein, but only the 55 kDa protein was ubiquitinated (Supplementary Figs. 6b and 7). In addition, the levels of both the 55 kDa and 35 kDa proteins were decreased after treatment of mouse GCPs with an siRNA against Slc7a11 (Supplementary Fig. 5c). These findings suggest that both the 55 kDa and the 35 kDa proteins are the Slc7a11 protein and that the 55 kDa protein is ubiquitinated.

There are several possible explanations for the opposite function observed for Jdp2 in GCPs and MEFs. First, the brain is more susceptible to oxidative stress than are other organs, and the cerebellar cell layers are particularly vulnerable to oxidative stress^57^. Thus, anti-oxidation molecules that control ROS homeostasis, including Jdp2, might be dysfunctional or reversed detecting during cerebellar development. Second, Nrf2-regulated GSH might be a major source of the anti-oxidation response detected during cerebellar development. However, GSH is increased in the brain at P12, but not at P5^32,33^. Thus, WT GCPs at P5 might exhibit lower Nrf2-mediated anti-oxidation activity. Third, the expression of Nrf2 was higher in *Jdp2*-KO GCPs than it was in WT GCPs; this is not the case in MEFs (see Fig. 4b, ref. ^43^). Fourth, the expression of small Mafs, including MafK, is higher in WT GCPs vs. *Jdp2*-KO GCPs, which is reversed in MEFs^43^. According to the results of ChIP and immunoprecipitation-western blotting assays performed in WT GCPs, Jdp2 is associated with Nrf2 or with MafK to reduce the occupancy of the Nrf2‒MafK activation complex on ARE; however, in the case of *Jdp2*-KO GCP2, only Nrf2 is associated with p21^Cip1^ to enhance ARE activity. It might be different in terms of function between p21^Cip1^ and MafK, which can couple with the same Nrf2, in GCPs and MEFs. Fifth, Jdp2 acts as a negative repressor of the ARE response in WT GCPs, whereas it functions as an activator in WT-MEFs because of its interaction with Nrf2‒MafK vs. Nrf2‒p21^Cip1^. Finally, ARE is mainly produced by the activation of *Slc7a11* in GCPs, to regulate GSH levels; however, in MEFs, the activation of *Ho1*, *Nqo1*, GSH is also detected. Although we do not know exactly why Jdp2 acts differently in GCPs and MEFs, ROS balance or Redox balance might act as a threshold factor. Here, we presented evidence that Jdp2 differs between these cell types. Therefore, different cell types, in this case GCPs and MEFs, may have different anti-oxidation mechanisms to regulate ROS homeostasis using Jdp2 as a modulator.

The interaction between Nrf2 and p21^Cip1^ might have provided maximal anti-oxidation ARE activity in *Jdp2*-KO GCPs because the formation of the Nrf2‒p21^Cip1^ complex was decreased in WT GCPs (Fig. 6a). Chen *et al*. reported that Nrf2 and p21^Cip1^ interact with each other, leading to the exclusion of the binding of Keap-1 to Nrf2 and an increase in Nrf2 protein stability and anti-oxidative responses^54^. Our sequential immunoprecipitation and western blotting experiments confirmed that the endogenous interaction between Nrf2 and p21^Cip1^ was stronger in *Jdp2*-KO GCPs than it was in WT GCPs, suggesting that the expression of Jdp2 in WT GCPs might repress the formation of the Nrf2‒p21^Cip1^ complex, although p21^Cip1^ was still present in the WT GCPs (Fig. 6h). Furthermore, the levels of both Nrf2 and p21^Cip1^ were increased in the absence of Jdp2 (Fig. 4b). As a result, the endogenous ROS level was reduced in *Jdp2*-KO GCPs, which might have enhanced their tolerance toward oxidative-stress-induced apoptosis (Fig. 2g).

In fact, the expression of apoptotic proteins, such as cytochrome C, Bad, Bax, and the cleaved caspases 3 and 8, was reduced in *Jdp2*-KO GCPs. The neuroprotection mediated by the insulin-like growth factor-1 and other neurotrophic factors is associated with the downregulation of Bax and Bad and with the induction of anti-apoptotic members of the Bcl-2 family^58^. p21^Cip1^ is a negative regulator of both p53-dependent and p53-independent apoptosis^59^. Here, we found that p21^Cip1^ was required for the prevention of ROS-induced cell death. Therefore, p21^Cip1^ plays a critical role in the Nrf2-regulated expression of *Slac7a11* and GSH production, thus preventing ROS-mediated apoptosis^60^. This is consistent with reports showing that the combined inhibition of GSH and *Slc7a11* synergistically enhances cell death^61,62^. It is also possible that Nrf2 targets different sets of oxidation-responsive genes by differentially interacting with Jdp2 or p21^Cip1^. In this regard, we found that *Gsr* and *Pgd* were upregulated in *Jdp2*-KO CGPs compared with WT GCPs (Fig. 4a). Moreover, the expression of Gsr^21,62^ and Pgd^63^ is reportedly upregulated by Nrf2. Together, these results imply that increased levels of the Nrf2‒p21^Cip1^ complex in the absence of Jdp2 may contribute to the upregulation of Gsr and Pgd.

In summary, we have demonstrated that the Nrf2‒p21^Cip1^ and Nrf2‒Jdp2 complexes differentially regulate the expression of *Slc7a11* to control ROS homeostasis and ROS-induced cell death in GCPs (Fig. 6h). The balance between the static amounts of Jdp2 and p21^Cip1^ and their interplay with Nrf2 in GCPs are critical for the ROS-induced neural cell death that occurs during cerebellar development. Further work on ROS balance mediated by Jdp2, Nrf2, and p21^Cip1^ in the control of other oxidative-stress-responsive genes, such as *Gsr*, *Pdh*, and *Cd44v*, is required to elucidate the precise developmental regulation of the cerebellum and of Purkinje cells.

## Materials and Methods

### Animals and cells

The animal welfare guidelines used for the care and use of laboratory animals were those published by the Animal Care Committee of the RIKEN BioResource Center (BRC) in Japan, the National Laboratory Animal Center (NLAC), and Kaohsiung Medical University in Taiwan. All animal experiments were performed in accordance with these approved guidelines. Mouse embryonic fibroblasts (MEFs) and 293 T cells were prepared as described^43,47,48^.

### Plasmids

The p*cDNA-Jdp2*, and p*cDNA-p21*^*Cip1*^ were obtained from RIKEN BRC as described previously^43,48,64^, amplified by polymerase chain reaction (PCR) and cloned into a pcDNA4 or a pcDNA3 vector using the respective restriction sites.

### Preparation and culture of primary GCPs

GCPs were prepared from the developing cerebellum as described previously^65,66^. P5‒P7 mice were decapitated, and their cerebellums were placed in cold Hanks glucose medium (Invitrogen-GIBCO, Thermo Fisher Scientific, Waltham, MA, USA) with Earle’s balanced salt solution (EBSS) containing papain (20 U/ml; Worthington, Lakewood, NJ, USA; LK003153), with 1 mM l-cysteine, 0.5 mM ethylenediaminetetraacetic acid (EDTA), and 0.05% DNase I at 37 °C for 15 min. Digestion was terminated by adding EBSS containing 0.2% ovomucoid (OM) and 0.1% bovine serum albumin (BSA). The tissues were pipetted repeatedly to obtain a suspension of single cells and then centrifuged at 200 *g* for 5 min. For discontinuous density-gradient centrifugation, the cell pellets were resuspended in 3 ml EBSS, 5 ml of BSA–OM inhibitor solution was added carefully onto the upper layer, and the cells were centrifuged at 70 *g* for 6 min^65^.

The cell pellets were resuspended in granule cell culture medium and incubated for 24 h in Neurobasal A medium containing 2% B-27 supplement, 100 U/ml penicillin, 100 μg/ml streptomycin, 2 mM glutamine, 5 μg/ml insulin, 100 μg/ml apotransferrin, and 16 μg/ml putrescine (all from Invitrogen-GIBCO) plus 1 mM sodium pyruvate, 100 μg/ml BSA, 30 μM N-acetyl cysteine (NAC), 62 ng/ml progesterone, 40 ng/ml sodium selenite, and 5 ng/ml epidermal growth factor (all from Merck, Darmstadt, Germany). The cells were then seeded into tissue culture dishes precoated with 50 μg/ml or 1.0 mg/ml poly-d-lysine (PDL; BD Biosciences, Billerica, MA, USA) and cultured for 1 day before the analysis. In some experiments, the cells were plated into eight-well cell culture dishes precoated with 1.0 mg/ml PDL^66^. Chemicals used for cell treatments, including 2,3,7,8-tertachlorodibenzo-p-dioxin (TCDD) or H_2_O_2_, were from Merck. The cells were incubated with TCDD or H_2_O_2_ for 2–24 h or 2 h, respectively, in culture medium without NAC. The viability of cells was determined using Trypan blue staining and calculated as described elsewhere^48,64^.

### Immunohistochemistry

For immunohistochemical staining, dewaxed slides were incubated with Peroxidase Block (3% H_2_O_2_) for 20 min and heated at 95 °C for 20 min in sodium citrate buffer (pH 6.0). After washing and blocking, the slides were incubated with the indicated antibodies against LacZ, caspase 3, Slc7a11, or Atoh-1 for 2 h and then incubated with the indicated mouse/rabbit probe horseradish peroxidase (HRP)-conjugated antibodies (BioTnA, Kaohsiung, Taiwan) for 30 min. After washing, the chromogen was developed by 3,3′-diaminobenzidine staining and the samples were counterstained with haematoxylin. The slides were rinsed with H_2_O and covered with resin-based mounting medium (BioTnA) after dehydration. Histopathological analysis was performed by the RIKEN BRC Experimental Animal Division in Japan and the NLAC, Taiwan. Tissue HE-staining was visualized using an Olympus CKX41 microscope (Olympus, Tokyo, Japan), and images were scanned and saved using a TissueFax microscope (TissueGnostics, Vienna, Austria).

### Immunocytochemistry

The tissue samples were stained with anti-LacZ, anti-caspase 3, or anti-Slc7a11 as described elsewhere^67,68^. GCPs were fixed in 4% formaldehyde for 10 min, washed with PBS, incubated with a blocking solution containing 10% fetal bovine serum and 0.1% Triton X-100 in PBS for 15 min to block non-specific binding sites, and incubated overnight with the anti-Atoh1 (1:400; Chemicon; AB5692), anti-LacZ antibody, or anti-Slc7a11 primary antibodies. After washing with PBS-Tween, the cells were incubated for 1.5 h with Alexa Fluor594-labeled goat anti-rabbit IgG (Life Technologies, Grand Island, NY, USA; A11037) and processed using 4′,6-diamino-2-phenylindole to visualize cell nuclei (1:3000; 5 mg/ml stock in dimethyl sulfoxide, Merck). Cells were mounted on slides using ProLong Gold Antifade Mountant (Molecular Probes, Thermo Fisher Scientific, Waltham, MA, USA; P36934), and cell immunofluorescence was visualized using an Olympus FV1000 confocal laser scanning microscope.

### Immunoprecipitation and western blotting

Immunoprecipitation and western blotting assays were conducted as described previously^48,64^. Briefly, primary cultured GCPs were homogenized in ice-cold N-PER Neuronal Protein Extraction Reagent (Thermo Fisher Scientific; #87792) according to the manufacturer’s instruction in the presence of Halt Phosphatase Inhibitor Cocktail (Thermo Fisher Scientific; #78440). Protein lysate aliquots (20 μg) were separated in NuPAGE 4‒12% Bis-Tris protein gels in 1.5 mm, 15-well dishes (Thermo Fisher Scientific; NP0335BOX) and transferred to 0.45 μm Immobilon-P transfer polyvinylidene difluoride (PVDF) membranes (Merck; IPVH00010) for 1 h at 100 V (fixed) at 10 °C using a TE22 Hoefer transfer system. Blots were stained with Ponceau S (Merck, P17170) to monitor the transferred protein amounts. The PVDF membranes were then probed with the primary and secondary antibodies according to the manufacture’s protocol (Research Resource Identifiers (https://www.rrids.org/). Bound antibodies were detected with HRP-labelled secondary antibodies using Immobilon^TM^ western chemiluminescent HRP substrate (EMD Millipore Co., Billerica, MA, USA; WBKL S0050) or SuperSignal^TM^ Western Plus, Chemiluminescent substrate (Thermo Scientific; #34579). Positive signals were analyzed using a ChemiDoc XRS_Plus instrument (Bio-Rad, Hercules, CA, USA). Immunoprecipitation was performed using antibody-coated protein-A/G beads, as described previously^69^. A summary of the antibodies is listed in Supplementary Table 1.

### Analysis of 7,8-dihydro-8-oxo-27-deoxyguanosine (8-oxo-dGuo), GSH, malonaldehyde (MDA), and cellular ROS

The concentration of 8-oxo-dGuo was measured using liquid chromatography–mass spectrometry, as described previously^70,71^. Total GSH and GSSG concentrations (mmol/mg protein) were measured using a GSH assay kit (Cayman Chemical Co.; Ann Arbor, MI, USA; 703002) and normalized against the protein concentration. The malondialdehyde (MDA) level was used as an indicator of lipid peroxidation was measured using the method of Uchiyama and Mihara^72^. To measure the intracellular ROS level, the ROS-Glo H2O2 assay (Promega) was used. After 2 h of treatment with H_2_O_2_ before cultivation for 24 h, the harvested cells were washed twice with Hank’s balanced salt solution (GIBCO), incubated with the ROS-Glo Detection Solution for 20 min, and the luminescence was measured using a GloMax luminometer (Promega).

### Cystine uptake

Following the basic protocol described by Bannai and Kitamura^73^, the cells were washed three times with prewarmed Na^+^-free PBS+G [10 mM PBS (137 mM choline chloride, 3 mM KCl), pH 7.4, containing 0.01% CaCl_2_, 0.01% MgCl_2_, and 0.1% glucose)] and then incubated in prewarmed uptake medium (0.5 ml) at 37 °C for 30 min. The uptake medium contained 20 μM cystine plus [^14^C]-cystine (0.2 μCi/ml) in Na^+^-free PBS (+) G. In some experiments, siRNA against Slc7a11, siRNA against p21^Cip1^_,_ or scrambled siRNAs were added at the indicated concentration 24 h before labelling and the cells were washed three times with prewarmed buffer. Uptake was terminated by rapidly rinsing the cells three times with ice-cold PBS, and the radioactivity in the cells was counted using a liquid scintillation counter as described elsewhere^73^.

### Measurement of intracellular cysteine

We followed to the basic protocol as described by Bannai’s group^73,74^. Briefly, cells were washed with PBS^(+)^G three times,and then incubated with 8 mM monobromobimane in 50 mM N-ethylmorpholine, pH 8.0 (100 μl), and 50 mM PBS^(+)^G containing 0.01% CaCl_2_, 0.01% MgCl_2_ and 0.1% glucose (100 μl), in the dark at room temperature for 30 min. Then, ten μl of 100% trichloroacetic acid was added to harvest the cells. The protein precipitate was removed by centrifugation at 3000 x g for 5 min, and aliquots were analyzed for cysteine-bimane adduct using high performance liquid chromatography (HPLC). The HPLC separation was performed on a steel column (4.6 × 100 mm) packed with 3-μm octadecylsilyl silica as reversed-phase. The fluorescence at 480 nm was monitored with the excitation wavelength at 394 nm. The materials were eluted with 9% (v/v) acetonitrile in 0.25% (v/v) acetic acid, pH 3.7 for 8 min from the column, and then 75% (v/v) acetonitrile in water for 5 min was run. The flow was done at 1 ml/min throughout the process^73,74^.

### Assays to measure apoptosis, caspase-Glo 3/7 activities, and annexin V level

The apoptotic cell population was measured using a DeadEnd Colorimetric TUNEL System (Promega) following the manufacturer’s protocol. Briefly, the cells were seeded at 5 × 10^5^ per well in six-well chamber slides and then fixed with 4% formaldehyde. The permeabilized cells were labelled with a terminal-deoxy-transferase reaction mixture for 1 h at 37 °C, the reaction was terminated by washing with 2× SSC buffer (0.3 M NaCl, 30 mM sodium citrate), and the cells were treated with streptavidin–HRP solution for 30 min at room temperature as indicated. The brown coloration developed by the diaminobenzene chromogen (Dako-Agilent, Santa Clara, CA, USA) was visualized. The apoptotic index was calculated as the percentage of positive cells in ≥500 cells at 400× magnification. Cell death was also determined by staining with Annexin V–fluorescein isothiocyanate and propidium iodide (PI) (Abcam, Cambridge, MA, USA; 14085). The cells were treated with the binding buffer with Annexin V and PI for 5 min in the dark and fixed in formaldehyde (4%) for 10 min, and the signals were measured on a FACSCalibur flow cytometer (BD Biosciences). The activities of caspase 3 and caspase 7 were measured using Caspase-Glo 3/7 Assay kits (Promega). The relative luminescence units were measured using a GloMax Luminometer (Promega).

### Statistical analysis

Quantitative variables are presented as the mean ± standard error (SEM) from triplicate experiments and additional replicates as indicated. The significance of differences was determined using Student *t* tests and one-way or two-way analysis of variance followed by Turkey’s test using GraphPad Prism 7.0. Differences with a *p* value < 0.05 were considered to be significant.

### Ethical approval and consent to participate

Animal experiments were approved by the Ethical Committee of the Kaohsiung Medical University, the National Laboratory Animal Center in Taiwan, and RIKEN BioResource Center in Japan and were conducted in accordance with their guidelines.

### Consent for publication

All authors have read the manuscript and agreed to its content. They are accountable for all aspects of the accuracy and integrity of the manuscript. The article is original, has not already been published in a journal, and is not currently under consideration by another journal.

## Supplementary information

Supplmentary Information.

## Data Availability

All relevant data are available on upon reasonable request from the authors. RNA sequencing data were deposited in the NCBI Bioproject Database (http://www.ncbi.nlm.nih.gov/bioproject) with the accession numbers SUB3541857, SUB3541902, SUB3541913, and SUB3541945. A reporting summary for this article is available as a Supplementary Information File).
