# Author Correction: *Jdp2*-deficient granule cell progenitors in the cerebellum are resistant to ROS-mediated apoptosis through xCT/Slc7a11 activation

**DOI:** 10.1038/s41598-020-66843-8

**Published:** 2020-06-12

**Authors:** Chia-Chen Ku, Kenly Wuputra, Kohsuke Kato, Wen-Hsin Lin, Jia-Bin Pan, Shih-Chieh Tsai, Che-Jung Kuo, Kan-Hung Lee, Yan-Liang Lee, Ying-Chu Lin, Shigeo Saito, Michiya Noguchi, Yukio Nakamura, Hiroyuki Miyoshi, Richard Eckner, Kyosuke Nagata, Deng-Chyang Wu, Chang-Shen Lin, Kazunari K. Yokoyama

**Affiliations:** 1 Graduate Institute of Medicine, Kaohsiung Medical University, 80708 Kaohsiung, Taiwan (R.O.C.); 2 Regenerative Medicine and Cell Therapy Research Center, Kaohsiung Medical University, 80708 Kaohsiung, Taiwan (R.O.C.); 3 Department of Infection Biology, Graduate School of Comprehensive Human Sciences, The University of Tsukuba, 305-8577 Tsukuba, Ibaraki Japan; 4 National Laboratory Animal Center, National Applied Research Laboratories (NARLabs), Xinshi Dist., 74147 Tainan, Taiwan (R.O.C.); 5 National Laboratory Animal Center, National Applied Research Laboratories (NARLabs), Nangang Dist., 11599 Taipei, Taiwan (R.O.C.); 6 Welgene Biotech., Inc., 11503 Taipei, Taiwan (R.O.C.); 7 School of Dentistry, Kaohsiung Medical University, 80708 Kaohsiung, Taiwan; 8 Saito Laboratory of Cell Technology, Yaita, 329-2192 Tochigi Japan; 9 Waseda Research Institute for Science & Engineering, Waseda University, 169-0051 Tokyo, Japan; 10 Cell Engineering Division, RIKEN BioResource Research Center, 305-0074 Tsukuba, Ibaraki Japan; 11 Department of Physiology, Keio University School of Medicine, Shinanaomachi, 168-8582 Tokyo Japan; 12 Departent of. Biochemistry & Molecular Biology, Rutgers New Jersey Medical School, The State University of New Jersey, 07-103 Newark, NJ USA; 13 Division of Gastroenterology, Department of Internal Medicine, Kaohsiung Medical University Hospital, 80708 Kaohsiung, Taiwan (R.O.C.); 14 Department of Biological Sciences, National Sun Yat-sen University, 80424 Kaohsiung, Taiwan (R.O.C.); 15 Department of Molecular Preventive Medicine, Graduate School of Medicine, The University of Tokyo, 113-8655 Tokyo, Japan; 16 Present Address: Founder of Gecoll Biomedicine Co. Ltd., Xinshi Dist., 744, Tainan, Taiwan (R.O.C.)

Correction to: *Scientific Reports* 10.1038/s41598-020-61692-x, published online 18 March 2020

In Figure 4B the relative intensity for the Nrf2 protein in Jdp2 cells was incorrectly given as 0.73. The correct Figure 4B appears below as Figure 1.

**Figure 1:**
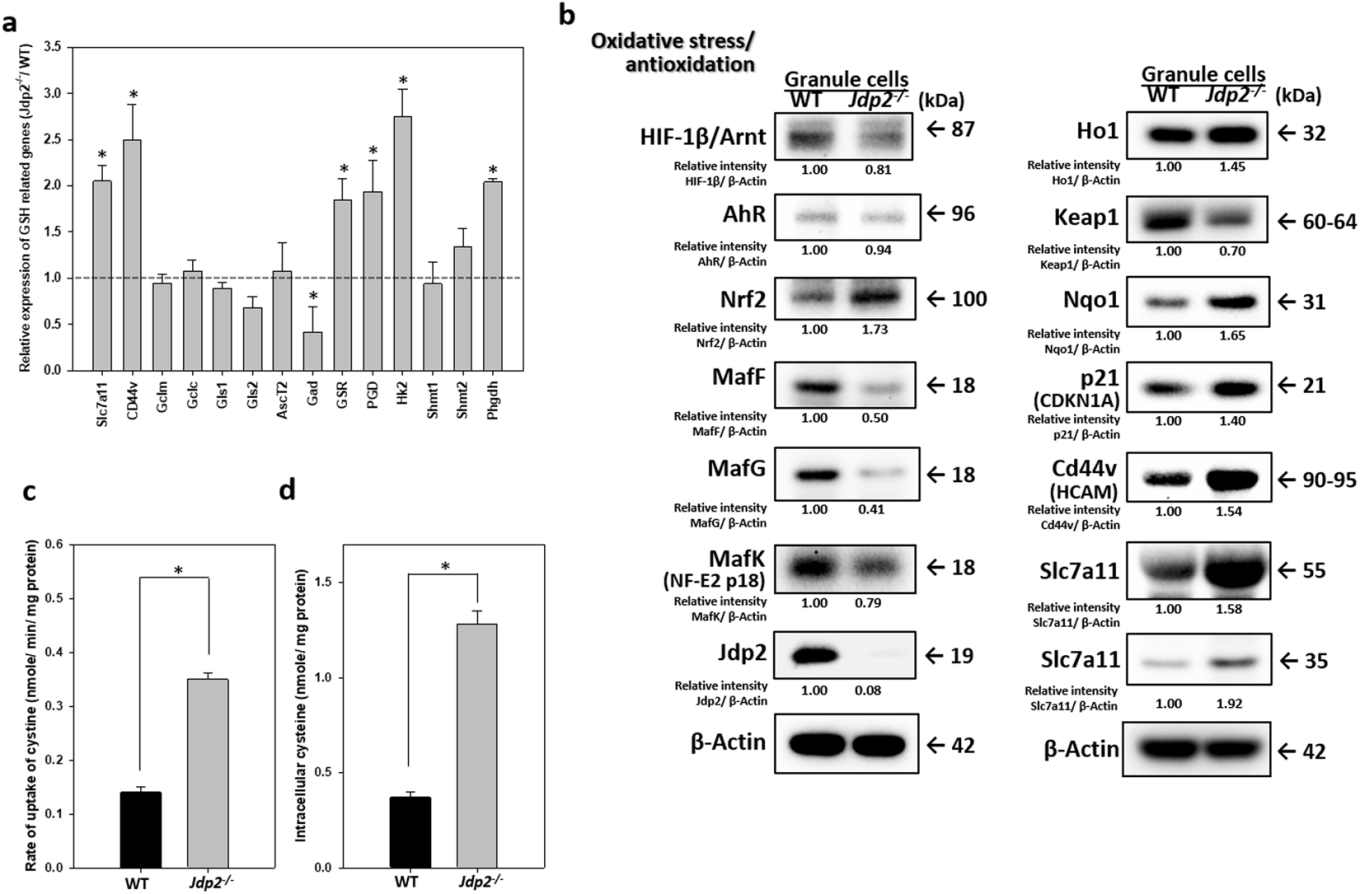
.

Additionally, the Supplementary Information file that accompanies this Article contains an error in Supplementary Figure S7 4B on page 18 where the Nrf2 protein in Jdp2 cells was incorrectly given as 0.73, 0.63 and 0.63 in the respective panels. The correct Figure S7 4B appears below as Figure 2.

**Figure 2:**
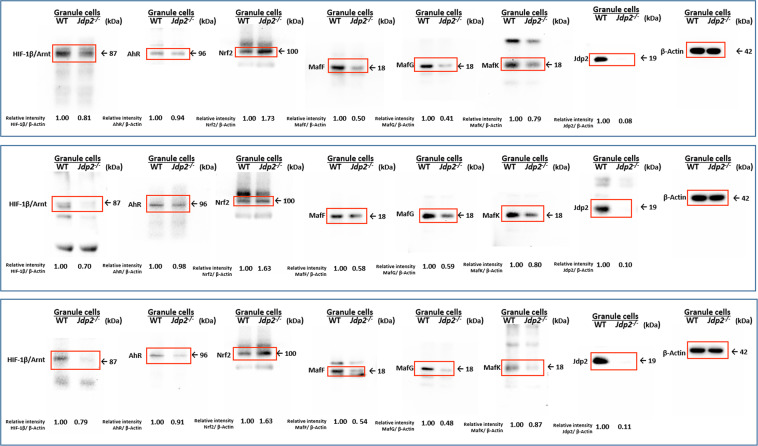
.

