# Supplementary material for: Jdp2-deficient granule cell progenitors in the cerebellum are resistant to ROS-mediated apoptosis through xCT/Slc7a11 activation
Source: Sci Rep. 2020 Mar 18;10:4933. doi: 10.1038/s41598-020-61692-x (PMC7080836; doi:10.1038/s41598-020-61692-x)
Supplement: Supplementary file 1 — Supplmentary Information. [file 41598_2020_61692_MOESM1_ESM.pdf]

Supplementary Information

Jdp2-deficient granule cell progenitors in the cerebellum are resistant to ROS-mediated apoptosis through xCT/Slc7a11 activation

Chia-Chen Ku[1,2,*], Kenly Wuputra[1,2,*], Kohsuke Kato[3], Wen-Hsin Lin[1,2], Jia-Bin Pan[1,2], Shih-Chieh Tsai[4,16] , Che-Jung Kuo[4], Kan-Hung Lee[5], Yan-Liang Lee[6], Ying-Chu Lin[7], Shigeo Saito[8,9], Michiya Noguchi[10], Yukio Nakamura[10], Hiroyuki Miyoshi[1,11], Richard Eckner[12], Kyosuke Nagata[3], Deng-Chyang Wu[2,13], Chang-Shen Lin[1,14,**], and Kazunari K. Yokoyama[1,2,15,**]

[1]Graduate Institute of Medicine, [2]Regenerative Medicine and Cell Therapy Research Center, [7]School of Dentistry, School of Medicine, Kaohsiung Medical University, Kaohsiung, Taiwan; [3]Department of Infection Biology, Graduate School of Comprehensive Human Sciences, the University of Tsukuba, Tsukuba, Japan; [4]National Laboratory Animal Center, National Applied Research Laboratories (NARL), Xinshi Dist., Tainan, Taiwan; [5]National Laboratory Animal Center, National Applied Research Laboratories (NARL), Nangang Dist., Taipei, Taiwan; [6]Welgene Biotech., Inc., Taipei, Taiwan; [8]Saito Laboratory of Cell Technology, Yaita, Tochigi, Japan; [9]Waseda Research Institute for Science & Engineering, Waseda University, Tokyo, Japan; [10]Cell Engineering Division, RIKEN BioResource Research Center, Tsukuba, Ibaraki, Japan;[11]Department of Physiology, Keio University School of Medicine, Shinanaomachi, Tokyo, Japan; Aug. 2019, Deceased; [12]Departent of. Biochemistry & Molecular Biology, Rutgers New Jersey Medical School, The State University of New Jersey, Newark, NJ, USA; [13]Division of Gastroenterology, Department of Internal Medicine, Kaohsiung Medical University Hospital, Kaohsiung, Taiwan; [14]Department of Biological Sciences, National Sun Yat-sen University, Kaohsiung, Taiwan; [15]Department of Molecular Preventive Medicine, Graduate School of Medicine, The University of Tokyo, Tokyo, Japan; [16]Present address; Founder of Gecoll Biomedicine Co. Ltd., Xinshi Dist., Tainan, Taiwan.

ORCID of KK. Yokoyama; 0000-0001-8508-7582; CS Lin; 0000-0001-7415-2187

*These authors contributed equally to this work.
**Chan-Shen Lin and Kazunari K. Yokoyama in Graduated Institute of Medicine, Kaohsiung Medical University, 100 Shih-Chuan 1st Road, San-Ming District, Kaohsiung 807, Taiwan; Phone; 886-7312-1101, ext. 2137, 2729; FAX; 886-7313-3849; e-mail; changshen.lin@gmail.com and kazu@kmu.edu.tw. These authors equally supervised this work.

1. **Methods section**
2. **Supplementary figure legends**

1. Methods section

Cells. Mouse astrocyte cell line CRL-2535-C8S was a gift from Dr. L-C Wang (Chang Gung University, Taiwan[1].

Generation and characterization of *Jdp2*-KO mice. The strategy to produce the *Jdp2*–KO mouse was as described previously[2,3]. A targeting vector (pTV-2) was generated by subcloning a 5.5-kbp DNA fragment, excised by *Eco*RV and *Xba*I from the 5'-upstream region of *Jdp2* gene, and a 2.3-kbp DNA fragment, excised by *Pst*I and *Sal*I from intron 1, into the pPNT vector[4], to serve as the 5'-long arm- and 3'-short arm-homologous regions, respectively. The strategy for knockout of the gene for *Jdp2* was described elsewhere[2,3]. The *Not*I-linearized targeting vector DNA was electroporated into E14tg2a ES cells[5] and cells were selected in the presence of 200 µg/ml G418 (Sigma-Aldrich Co., St. Louis, MO, USA). Southern blots of *Xba*I-digested genomic DNA from colonies of drug-resistant ES cells were hybridized with an external probe derived from intron 1. Resistant colonies were subjected to Southern analysis with a [^{32}P]-labeled DNA fragment for hybridization outside the right arm, which recycled an *Xba*I band of 3.4 kb in the case of wild-type gene and of 4.7 kb in the case of the correctly targeted allele. Two ES clones harboring the disrupted gene were injected into C57BL/6 blastocysts for generation of chimeric mice. The progeny of mating between chimeras and C57BL/6 mice were genotyped by Southern blot hybridization and, also, by PCR with two primer pairs: (1) a forward primer (5’-TATGGGTGATGACCTGCTGT-3’) from the 5’ upstream region of the promoter and a reverse primer (5'-CAGGATCTCGCAAGCTTGTT-3') from exon 1, which amplified a fragment of 788 bp from the wild-type gene; and (2) a specific reverse primer (5'-TCCTCGTGCTTTACGGTATC-3') from the neomycin-resistance cassette and the common forward primer, which amplified a fragment of 593 bp that was specific for the targeted allele. The heterozygous mice, with a mixed C57BL/6 × 129 background, were bred to generate WT, *Jdp2*$^{+/-}$ and *Jdp2*$^{-/-}$KO mice (KO) for subsequent analyses. We generated chimeric mice by injecting clones of targeted ES cells into blastocytes from C57BL/6J mice. Chimeric males were then mated with C57BL/6J females to produce an F1 generation. Inter-crosses between heterozygotes yielded homozygous mutants at the expected ratio. “Knock-out (KO)” mice carried a disrupted allele of the *Jdp2* gene, in which a 3,048-bp DNA fragment flanked by *Xba*I and *Pst*I sites had been replaced by a cassette from pGKpro-Neo-polyA. This region includes three possible sites of initiation of transcription (GenBank, AB034697, BC019780

and AB077438) of the *Jdp2* gene[6]. We detected WT and mutated *Jdp2* alleles by Southern blotting and PCR-based genotyping. The genotype study also demonstrated that the F2 offspring produced by mating heterozygous males and females conformed to Mendel's law. We found no abnormalities with regard to reproduction and life span in the *Jdp2* hetero- or homo-KO mice under normal breeding conditions, with the exception of tail length. The phenotype and behavior in our *Jdp2* KO mice resembled that of the WT, but tails were shorter and the ratio of tail length to body length was smaller than that of WT control mice. These observations were consistent with those from KO mice with a defect in the coding region of the gene for JDP2 (exon 2 KO).

Preparation of *Jdp2*-promoter-*Cre* mice. The DNA insert contained a 5'-end of the promoter region of *Jdp2* gene (2.5 kb), the initiation site of transcription preceding the ATG codon of *Jdp2* mRNA, a nuclear localization signal (NLS) fragment of the *Cre* gene, internal ribosome entry site DNA fragment, and the NLS-LacZ (β-galactosidase) DNA followed by an SV40 polyadenylation motif (polyA)[7]. The *Swa*1 linearized DNA was used for microinjection into fertilized FVB/N eggs. Twenty-five transgenic mouse lines (Fo) were obtained and further crossed with ROSA26R reporter[8] or ZEG (TCTB-βgeo–Green Fluorescein Protein) reporter[9] mice. Functional F1 offspring was characterized by X-gal staining or GFP, and the genotype of these F1 founders was examined by PCR. PCR genotyping was performed with the following three primers: Primer1, 5'-GGGTTAAGTGGAATCAGTTCTGCTC-3'; Primer 2, 5'-GGTTCAGGGGGAGGTGTGGGAGG-3' (SV40 polyA); Primer 3, 5'-GGAAGGCGATCCCATAGGAAGAG-3'. The sizes of the WT (Primers 1 and 3) and mutant DNA fragments (Primer 1 and Primer 3) were 688 and 420 bp, respectively. C57BL6/J × 129v, 129v and C57BL6/J congenic backgrounds were available from the RIKEN BioResource Center (RIKEN BRC) at Tsukuba, Japan, and National Laboratories of Animal Center (NLAC) in Taipei, Taiwan.

Small interfering (si) RNAs. The siRNAs targeting the expression of *p21^{Cip1}* and *Slc7a11* and a scrambled siRNA were obtained from Ambion (Thermo Fisher Scientific; s63812) and Sigma-Aldrich (St Louis, MO, USA; NM_001111-97 and NM_007669). TRCN 000081974

proved to be the most effective for GCPs. The sequence of the *Jdp2* siRNAs are available from the authors on request. GCPs were incubated for 24 h to adhere to the flask and were then transfected with siRNA using siPORT transfection agent (Ambion) in six-well plates according to the manufacturer's protocols. Silencing was confirmed by western blotting or quantitative polymerase chain reaction (qPCR) using appropriate antibodies or the primers after incubation for 24 h. A summary of the siRNAs sequences is listed in Supplementary Table 2.

RNA isolation and reverse transcription (RT)-quantitative polymerase chain reaction (qPCR). Total RNA was extracted from various tissues including embryos, GCPs and MEFs of WT and *Jdp2*-KO mice by TRIzol (Invitrogen) according to the manufacturer's protocols[10]. Real-time qPCR was carried out by the Thermo Fisher Scientific Fat SYBER™ Green Mix for relative quantification using an ABI 7500 Fast Real-Time PCR system (Applied Biosystems, Foster City, CA, USA) using the standard protocol. The qPCR primers were designed using the GENETYX-Mac software (version 14; Hitachi Software, Tokyo, Japan), and the sequences are listed in Supplementary Table 3.

Transfection and luciferase reporter assays. The ARE–luciferase reporter pGL4-hQR25–Luc containing an ARE from the human *NQO1* promoter (nucleotides −471 to −447) and the mouse *Slc7a11* promoter–Luc (pGL3-4.7) containing an ARE have been described previously[11,12] [a kind gift from H. Sato, Niigata University, Niigata, Japan]. WT and *Jdp2*-KO GCPs (5×10^6 cells) were plated into each well of 24-well plates and cultured for 24 h. The cells were cotransfected with 400 ng of pGL4-hQR25–Luc or pGL3-4.7 (human xCT–luciferase) and 100 ng of the pGL4 or pGL3 plasmid encoding *Renilla* luciferase (Promega Co., Madison, WI, USA) as an internal control for transfection efficiency using Effectene Transfection Reagent kits (Qiagen, Hilden, Germany) or Lipofectamine 2000 (Invitrogen, Grand Island, NY, USA) as described in the manufacturers' protocols. For forced expression and knockdown, cells were cotransfected with pcDNA3 vectors encoding $p21^{Cip1}$ or Jdp2, or siRNA against $p21^{Cip1}$ or Slc7a11 and then cultured for 24 h. The total amount of transfected DNA was kept constant at 500 ng/well by adding a pcDNA3 control vector. After 24 h of incubation after transfection, the firefly and *Renilla* luciferases activities were measured in a

luminometer (Berthold Technologies, Bad Wildbad, Germany) using the Dual-Luciferase Reporter Assay System (Promega) as described elsewhere[2,3,11]. The relative transcription activity was calculated as the fold induction relative to the control cells.

Chromatin immunoprecipitation (ChIP) analysis. The ChIP assay was performed using GCPs from WT and *Jdp2*-KO mice as described[11]. WT and *Jdp2*-KO GCPs (5×10^7 cells) were fixed with 1% formaldehyde (Sigma, F8775) for 10 min, and cross-linking was quenched by adding glycine (125 mM) and incubated on ice for 5 min. GCPs were centrifuged at 500 g, for 3 min at 4 °C and the pellet was washed in ice cold PBS. The, GCPs were lysed by the RIPA buffer. Lysed cells were sonicated by Bioruptor Pico device (Diagenode Com. B01060010) for 3 min 7 cycles (30s ON/30s OFF) and centrifuged at 12,500g, for 10 min at 4 °C. The chromatin were divided and transferred to the new tubes and incubated with antibodies as indicated (ChiP grade; 600 μg chromatin was incubated with 3 μg of anti-Nrf2, 2 μg of anti-Jdp2, 1 μg of anti-MafK, 3 μg of anti-p21^{Cip1}, 2 μg of anti-HDAC1, 2, and 3, 3 μg of anti-CBP and 4 μg of IgG) at 4 °C overnight on a rotating wheel. 40 μl of protein A and G Dynabeads were added and incubated for another 2 h. The complexes were washed with binding buffer (10 mM HEPES, pH 7.9, 10 mM Tris-HCl, pH 7.9, 12.5% glycerol, 0.25% NP-40, 0.5% Triton X-100, 0.24 M NaCl, 0.75 mM $MgCl_2$, 1.1 mM EDTA, and protease inhibitor mixture) twice, and then washed with Tris-EDTA buffer (10 mM Tris-HCl, pH 7.9, and 1 mM EDTA) twice again. After digestion of protein–DNA complexes with proteinase K (Sigma-Aldrich) at pH 6.8, DNA was extracted with phenol-chloroform and precipitated in ethanol and analyzed by real-time PCR using a Power SYBR Green Master Mix (Invitrogen). The PCR conditions were one cycle at 50 °C for 2 min and one cycle at 95 °C for 10 min followed by 40 cycles at 95 °C for 15 s and 60 °C for 60 s. The primers used in this experiment were as follows: SLC7a11-ARE, forward, 5'-ATTGAGCAACCCACAGGC-3′ and reverse, 5′-CTCGTGGAAGGCTCCGTA-3′; N-site, forward, 5′-TGCCACATGCTGTTGCTT-3′ and reverse, 5′-TTTCCCCTCCTCTGGGAC-3′.

RNA sequencing. RNA sequencing was performed using a Genome Analyzer IIX System (Illumina, San Diego, CA, USA) and the 50 bp single-end protocol by Welgene Biotech

(Taipei, Taiwan) as described[13,14]. RNA sequencing data were deposited in the NCBI Bioproject Database (http://www.ncbi.nlm.nih.gov/bioproject) with the accession numbers SUB3541857, SUB3541902, SUB3541913, and SUB3541945).

References

1 Ahlgren-Beckendorf, J. A., Reising, A. M., Schander, M. A., Herdler, J. W. & Johnson, J. A. Coordinate regulation of NAD(P)H:quinone oxidoreductase and glutathione-S-transferases in primary cultures of rat neurons and glia: role of the antioxidant/electrophile responsive element. *Glia* **25**, 131-142 (1999).

2 Pan, J. *et al.* Suppression of cell-cycle progression by Jun dimerization protein-2 (JDP2) involves downregulation of cyclin-A2. *Oncogene* **29**, 6245-6256, doi:10.1038/onc.2010.355 (2010).

3 Nakade, K. *et al.* JDP2 suppresses adipocyte differentiation by regulating histone acetylation. *Cell death and differentiation* **14**, 1398-1405, doi:10.1038/sj.cdd.4402129 (2007).

4 Tybulewicz, V. L., Crawford, C. E., Jackson, P. K., Bronson, R. T. & Mulligan, R. C. Neonatal lethality and lymphopenia in mice with a homozygous disruption of the c-abl proto-oncogene. *Cell* **65**, 1153-1163, doi:10.1016/0092-8674(91)90011-m (1991).

5 Niwa, H., Miyazaki, J. & Smith, A. G. Quantitative expression of Oct-3/4 defines differentiation, dedifferentiation or self-renewal of ES cells. *Nature genetics* **24**, 372-376, doi:10.1038/74199 (2000).

6 Tsai, M. H., Wuputra, K., Lin, Y. C., Lin, C. S. & Yokoyama, K. K. Multiple functions of the histone chaperone Jun dimerization protein 2. *Gene* **590**, 193-200, doi:10.1016/j.gene.2016.03.048 (2016).

7 Chiou, S. S. *et al.* Control of Oxidative Stress and Generation of Induced Pluripotent Stem Cell-like Cells by Jun Dimerization Protein 2. *Cancers* **5**, 959-984, doi:10.3390/cancers5030959 (2013).

8 Soriano, P. Generalized lacZ expression with the ROSA26 Cre reporter strain. *Nature genetics* **21**, 70-71, doi:10.1038/5007 (1999).

9 Novak, A., Guo, C., Yang, W., Nagy, A. & Lobe, C. G. Z/EG, a double reporter mouse line that expresses enhanced green fluorescent protein upon Cre-mediated excision. *Genesis (New York, N.Y. : 2000)* **28**, 147-155 (2000).

10 Kawasaki, H. *et al.* ATF-2 has intrinsic histone acetyltransferase activity which is modulated by phosphorylation. *Nature* **405**, 195-200, doi:10.1038/35012097 (2000).

11 Tanigawa, S. *et al.* Jun dimerization protein 2 is a critical component of the Nrf2/MafK complex regulating the response to ROS homeostasis. *Cell death & disease* **4**, e921,

doi:10.1038/cddis.2013.448 (2013).

12 Sato, H. *et al.* Transcriptional control of cystine/glutamate transporter gene by amino acid deprivation. *Biochem Biophys Res Commun* **325**, 109-116, doi:10.1016/j.bbrc.2004.10.009 (2004).

13 Kuo, K. K. *et al.* Positive Feedback Loop of OCT4 and c-JUN Expedites Cancer Stemness in Liver Cancer. *Stem cells (Dayton, Ohio)* **34**, 2613-2624, doi:10.1002/stem.2447 (2016).

14 Wu, D. C. *et al.* Reprogramming Antagonizes the Oncogenicity of HOXA13-Long Noncoding RNA HOTTIP Axis in Gastric Cancer Cells. *Stem cells (Dayton, Ohio)* **35**, 2115-2128, doi:10.1002/stem.2674 (2017).

Supplementary Figure legends

Fig. S1. Comparative expression of Nrf2 and its related antioxidation proteins in GCPs from WT and *Jdp2*-KO mice were examined by western blotting in the presence and absence of NAC. After the 24 h exposure, the cells were harvested, proteins extracted, and IP-WBs was performed as described in Methods. The relative intensity of each band was calculated relative to β-actin (n = 3).

Fig. S2. Effect of TCDD on ROS activity and intracellular GSH level in WT and *Jdp2*-KO GCPs. WT and *Jdp2*-KO GCPs were cultured in the presence of 100 nM TCDD, harvested at 0 h, 2 h, 6 h, 16 h, and 24 h, and subjected to various assays such as ROS production (a) and intracellular GSH (b). Each value represents the mean ± SEM (n = 3); * $p < 0.05$.

Fig. S3. Comparative expression of cerebellum related and neural mRNAs. (a, b) RNA expression of genes encoding cerebellum (a) and Neural factors (b) was examined by RNA sequencing in GCPs from WT and *Jdp2*-KO mice (a; n = 2) and RT-qPCR (b; n = 3; mean ± SEM; * $p < 0.05$, two-tailed Student's *t* test). The expression in WT GCPs was set as 1.0 (except for *Oct4* and *Nanog* because their mRNAs were barely detected). Values represent the mean ± SEM (n = 3); $p < 0.05$.

Fig. S4. Schematic representation of proteins that generate GSH and prevent ROS production. Gsr; glutathione reductase, Pgd; 6-phosphoglycero dehydrogenase, Hk2; hexokinase 2,

Phdgh; phosphoglycerate dehydrogenase, Gclm; glutamate–cysteine ligase complex modifier subunit, Gclc; glutamate–cysteine ligase complex catalytic subunit, G6p; glucose-6-phosphate, Gpx4; glutathione peroxidase 4; 3-Pg; 3-phosphoglycerol; GSH; glutathione-SH.

Fig. S5. Effects of siRNAs against $p21^{Cip1}$ and Slc7a11 on $p21^{Cip1}$ protein expression, *Slc7a11* mRNA expression, and Slac7a11 protein expression. (a) Effect of siRNA$p21^{Cip1}$ on expression of $p21^{Cip1}$ protein. Western blot analysis was performed as described in Methods. (b) The mRNA expression of *Slc7a11* with or without siRNA1, siRNA2, and siRNA 3 against $p21^{Cip1}$. The value of gene-specific siRNA (–) in WT GCPs was set as 1.0. Each value represents the mean ± SEM (n = 3); * $p < 0.05$. (c) Effects of siRNA against Slc7a11 on Slc7a11 protein expression. GCPs from WT and *Jdp*2-KO mice were cultured in the presence of scrambled siRNA, siRNA#1, #2, and #3 against Slc7a11. The expression of Slc7a11 was evaluated using antibodies against Slc7a11 from different companies (ARG57998, bs-6883R, and CST #98051). The relative intensity of each band was calculated relative to β-actin (n = 3).

Fig. S6. Comparative studies of proteins immunoprecipitated with different antibodies against Slc7a11 (ARG57998, bs-6883R, and CST #98051). (a) Comparative expression of Slc7a11 55 kDa and 35 kDa proteins in GCPs from WT and *Jdp2*-KO mice were examined by western blot analysis. The cells lysates were extracted and analyzed by western blot analysis using different commercially available antibodies against Slc7a11. (b) The interaction of Slc7a11 55 kDa protein and 35 kDa protein was examined by IP-WBs as described in Methods. The first trapping was performed using ARG57998 and the bound proteins were blotted using ARG57998, bs-6883R, and CST #98051. The relative intensity of each band was calculated relative to β-actin (n = 3).

Fig. S7. Full-length western blot analysis used in this work. The molecular weight markers are listed. At least three trials of each protein were performed, and the values were calculated

relative to β-actin.

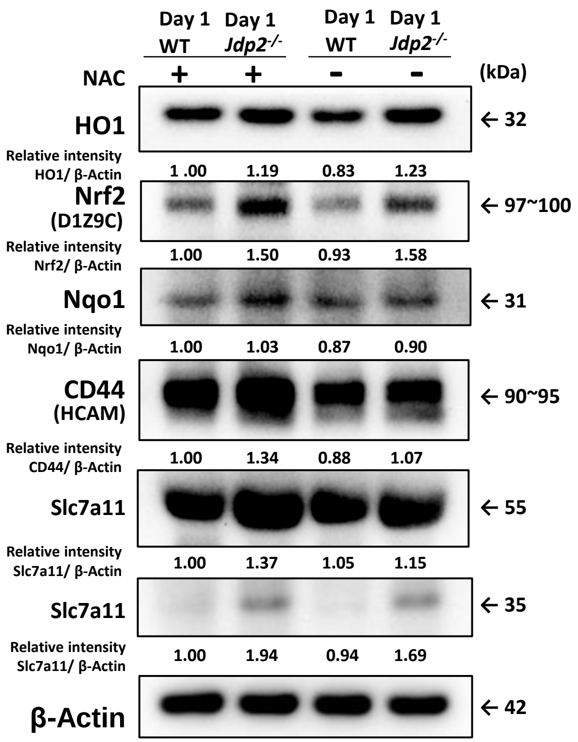

Supplementary Figure 1

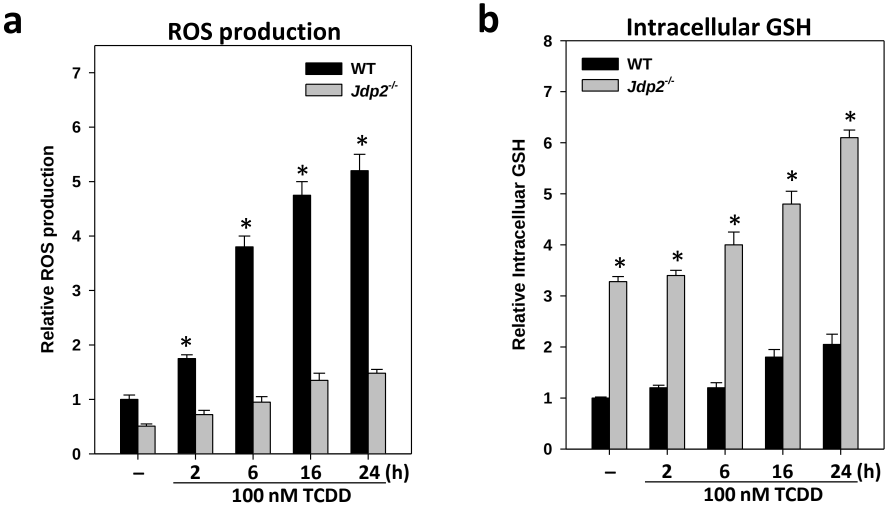

Supplementary Figure 2

a

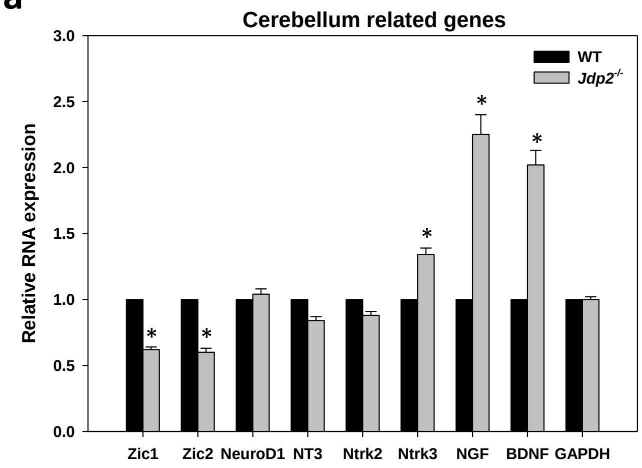

b

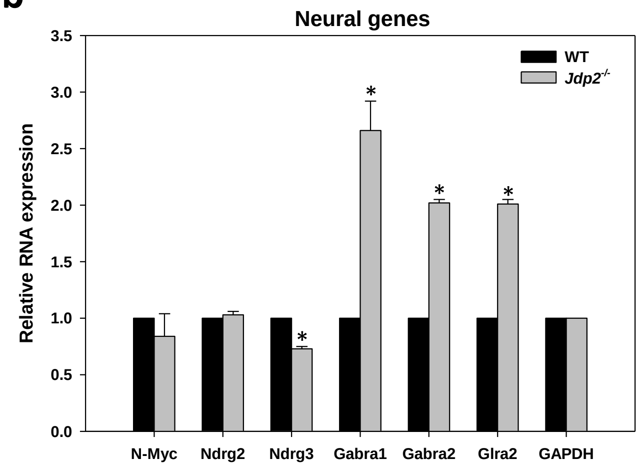

Supplementary Figure 3

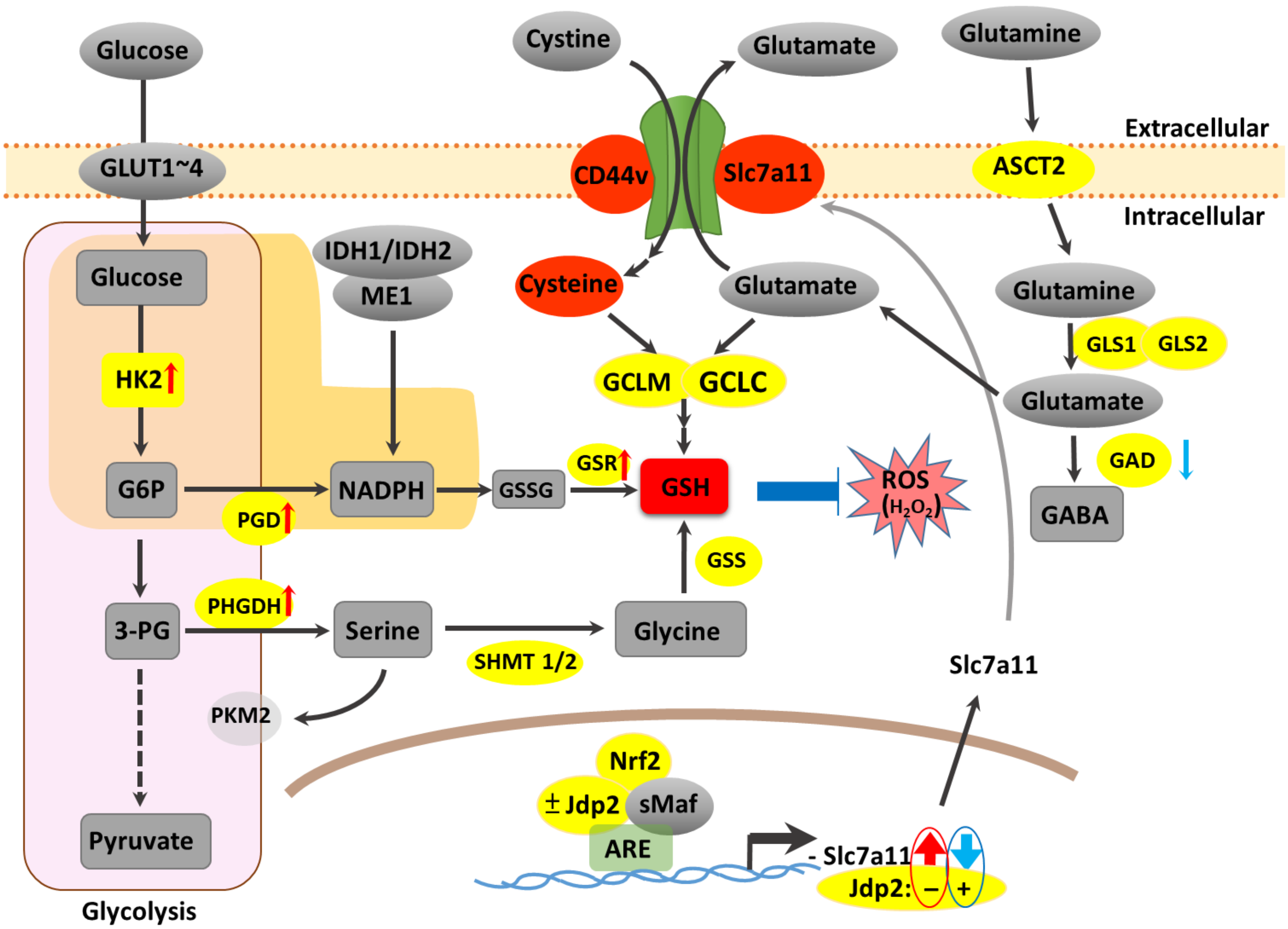

Supplementary Figure 4

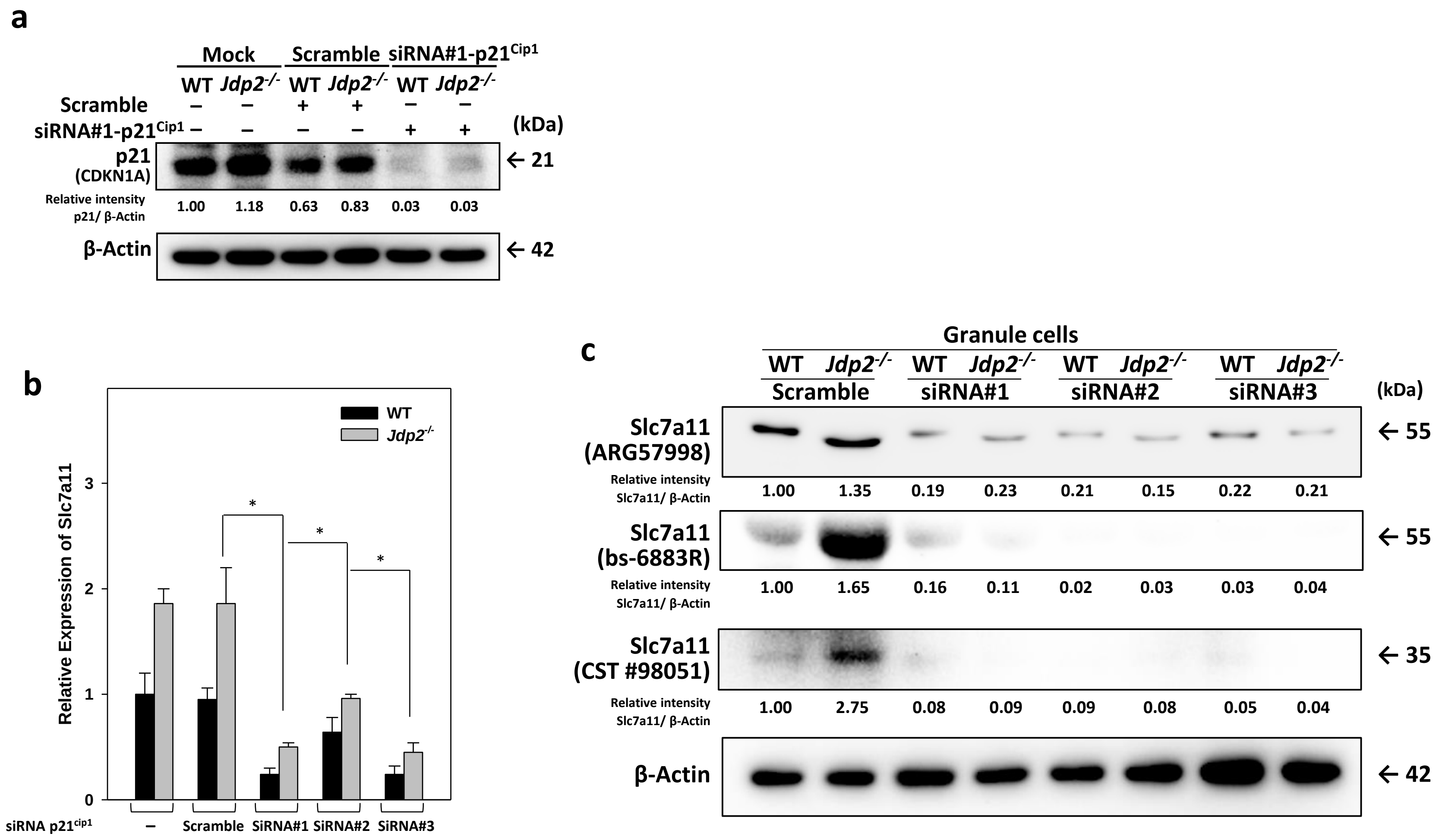

Supplementary Figure 5

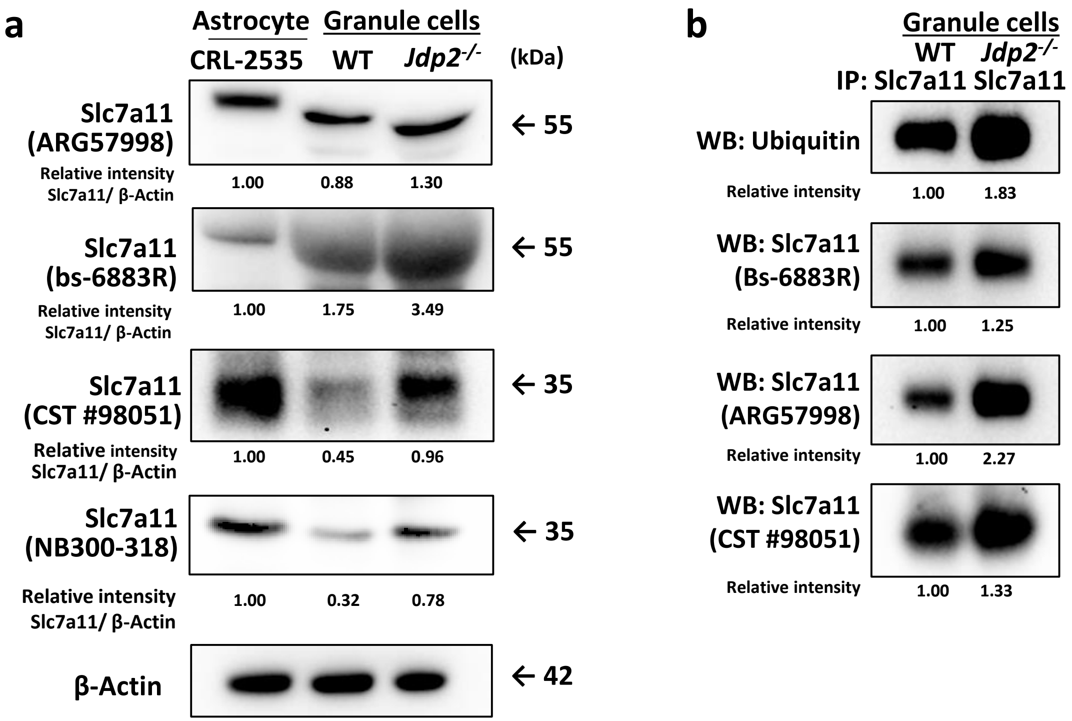

Supplementary Figure 6

Fig 1D

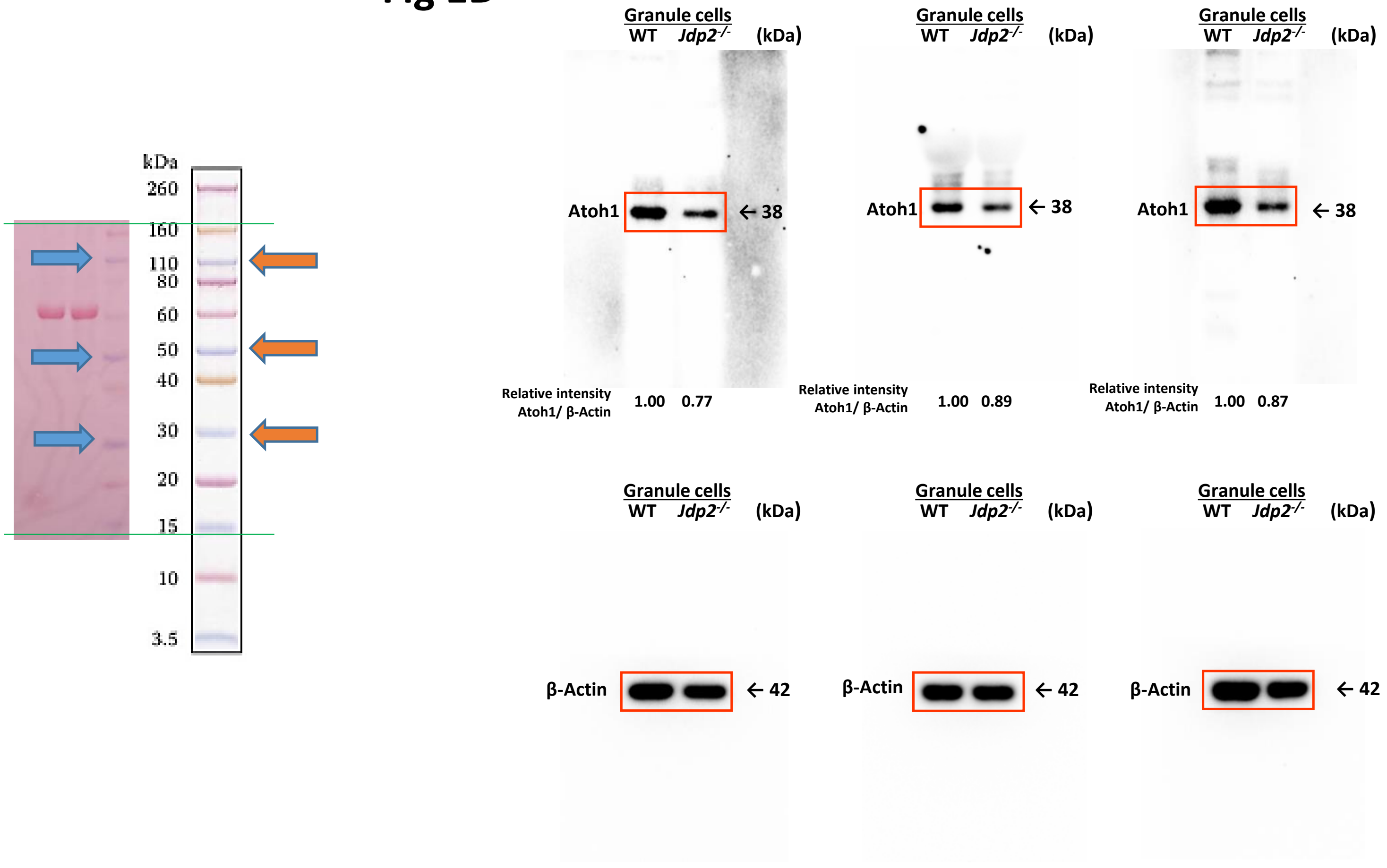

Supplementary Figure 7

Fig 2F

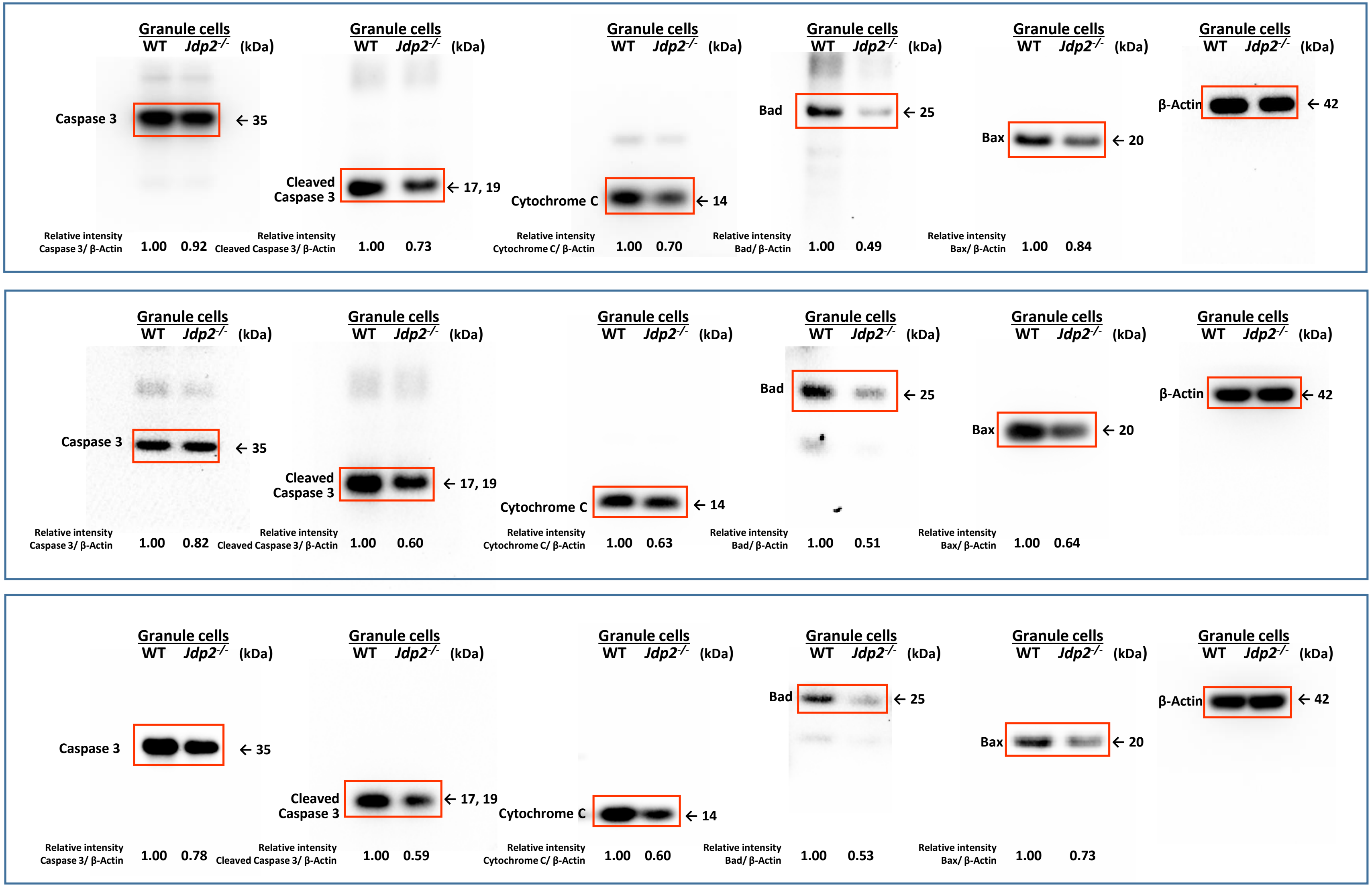

Supplementary Figure 7

Fig 4B

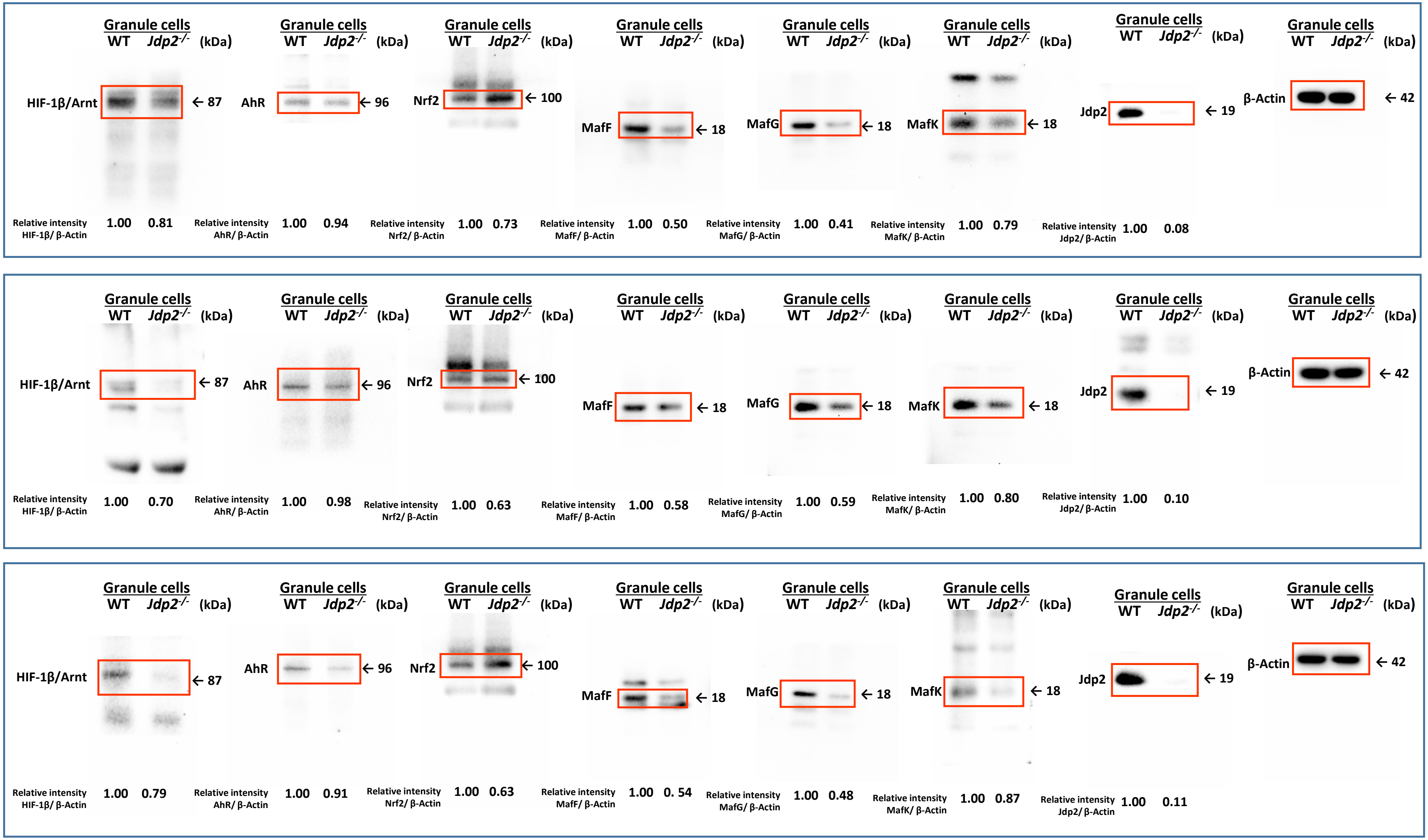

Supplementary Figure 7

Fig 4B

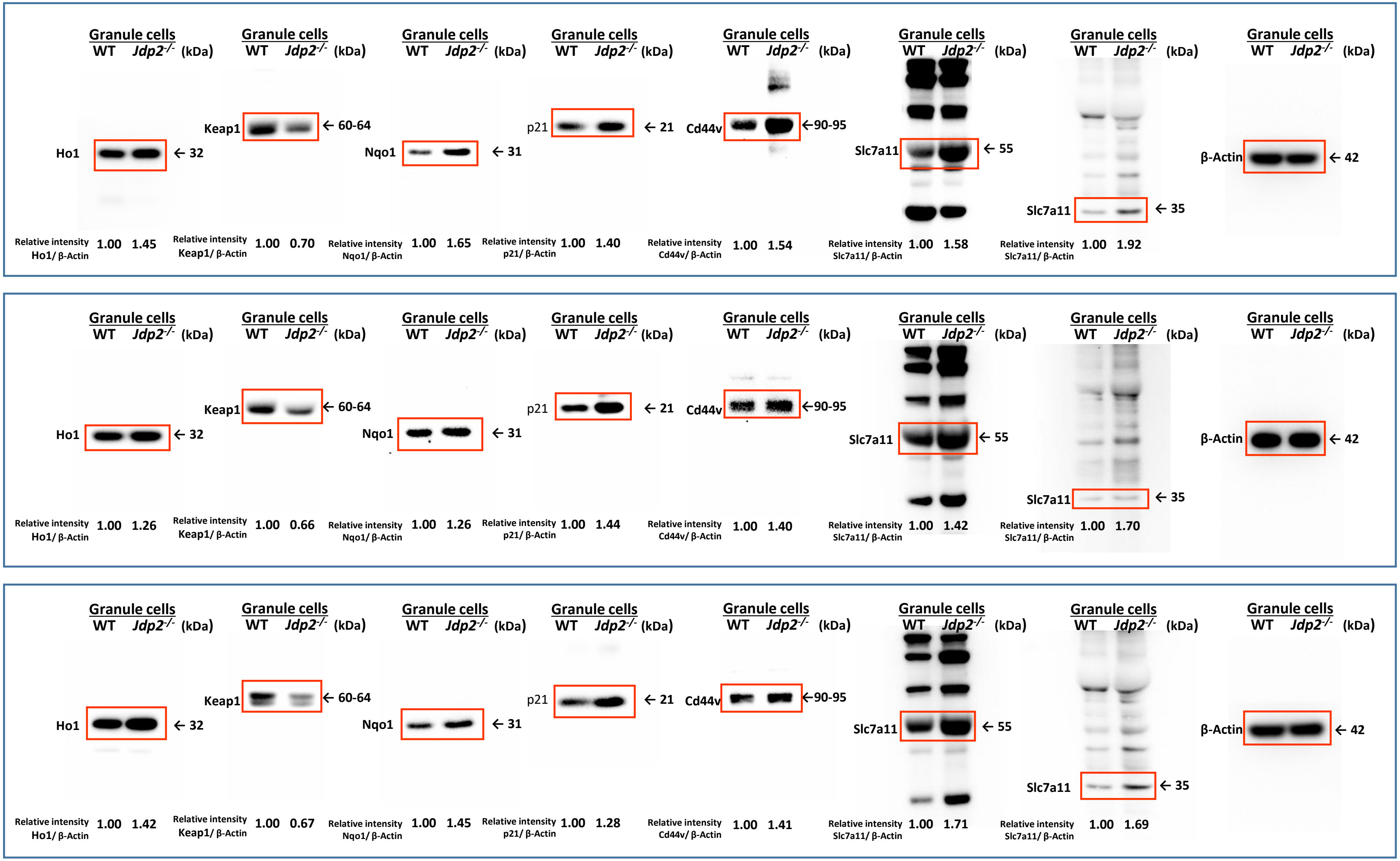

Supplementary Figure 7

Fig 6A

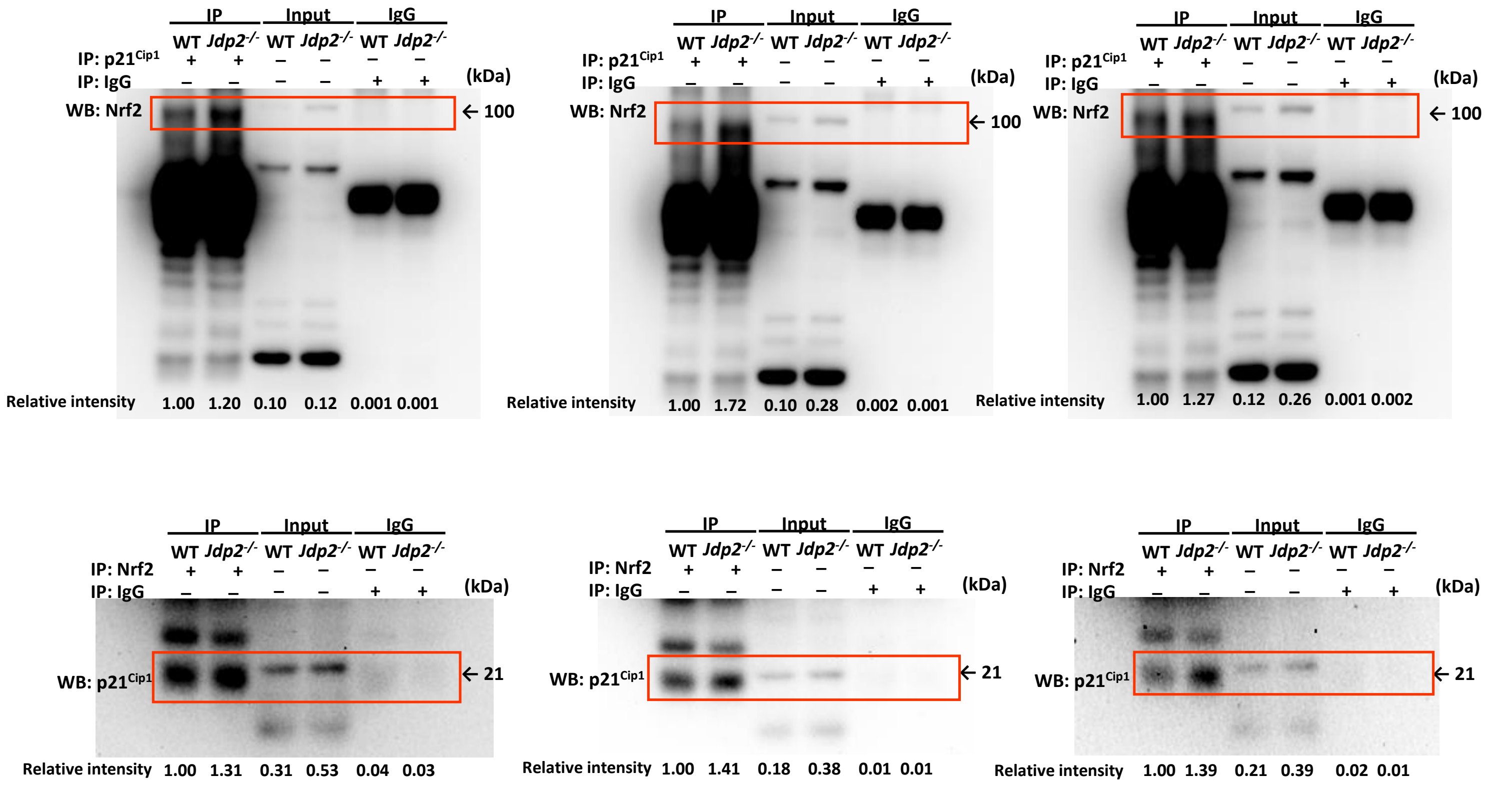

Supplementary Figure 7

SFig1

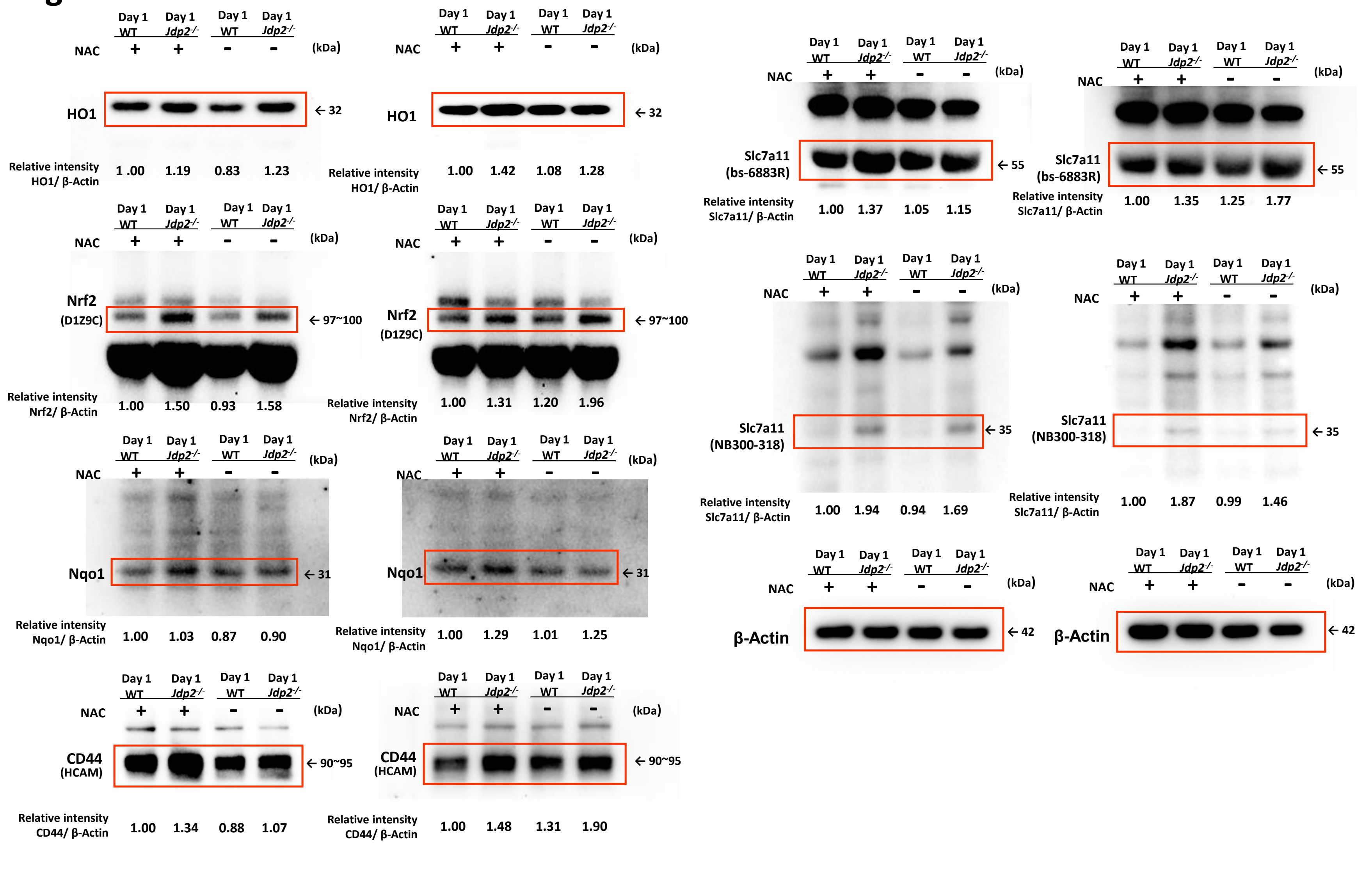

Supplementary Figure 7

SFig5A

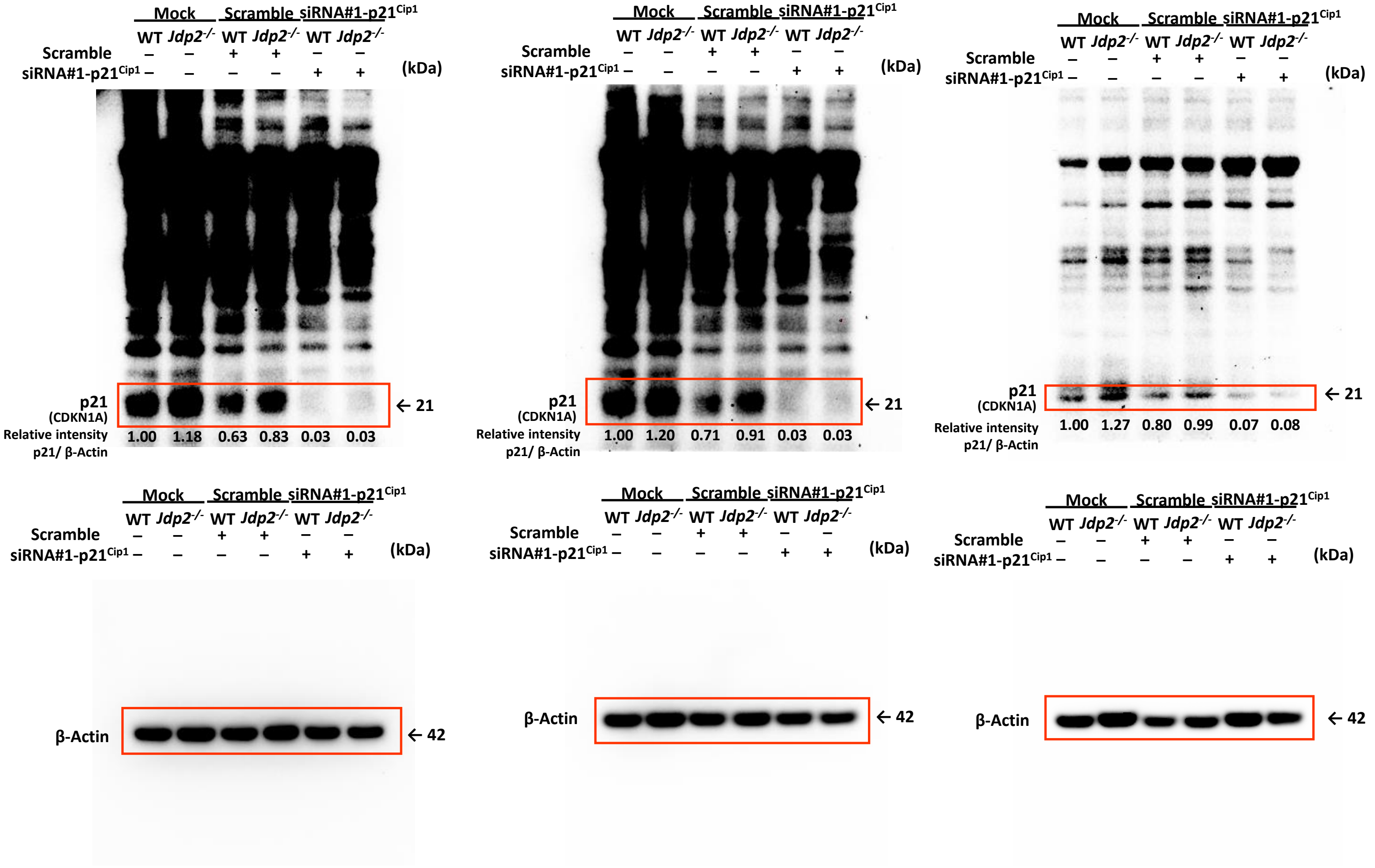

Supplementary Figure 7

SFig5C

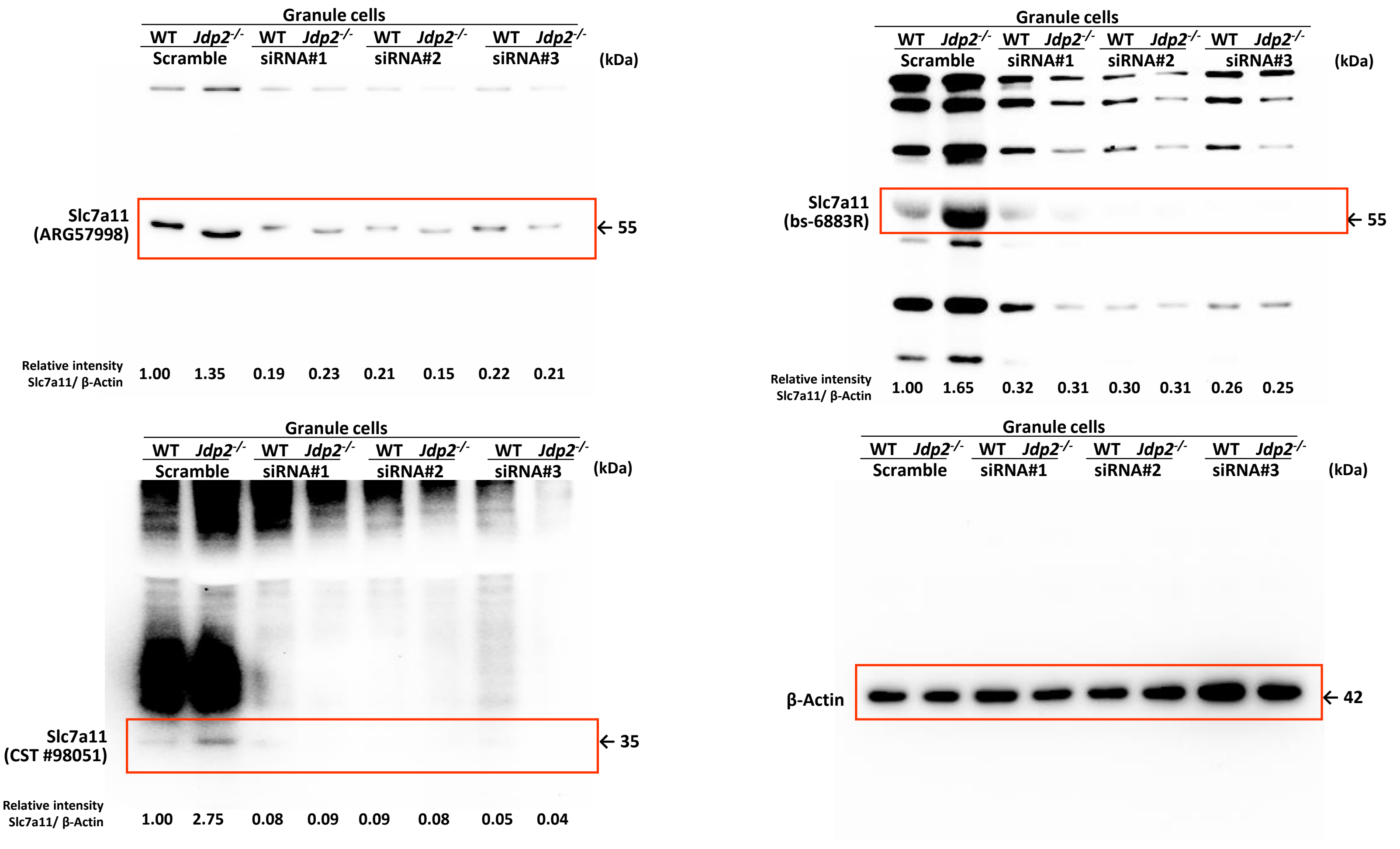

Supplementary Figure 7

SFig6A

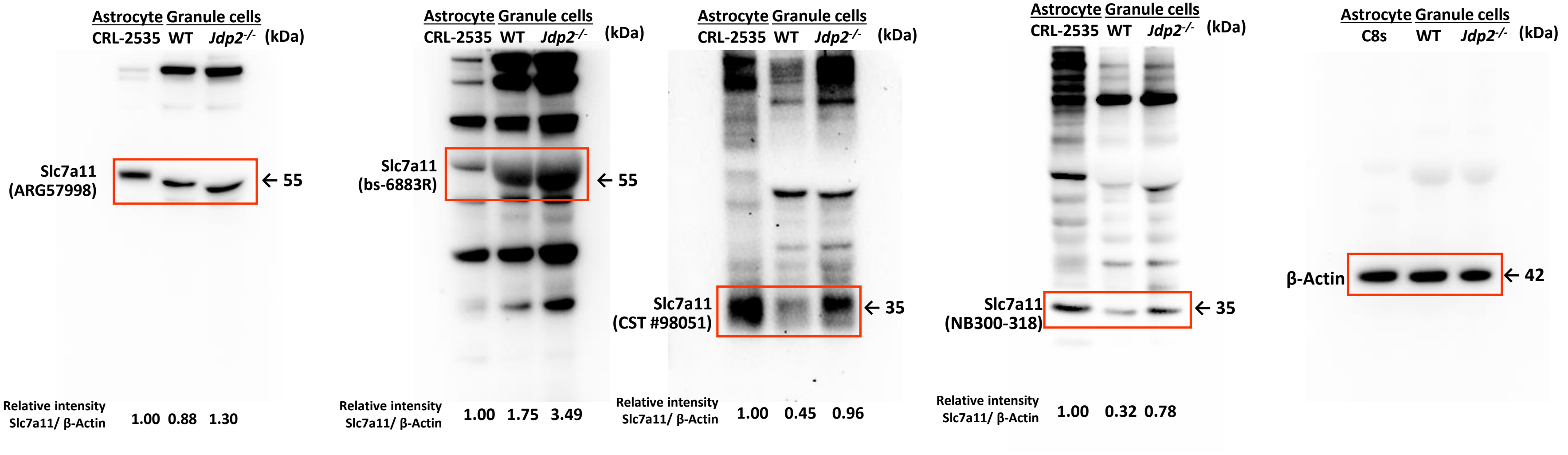

Supplementary Figure 7

SFig6B

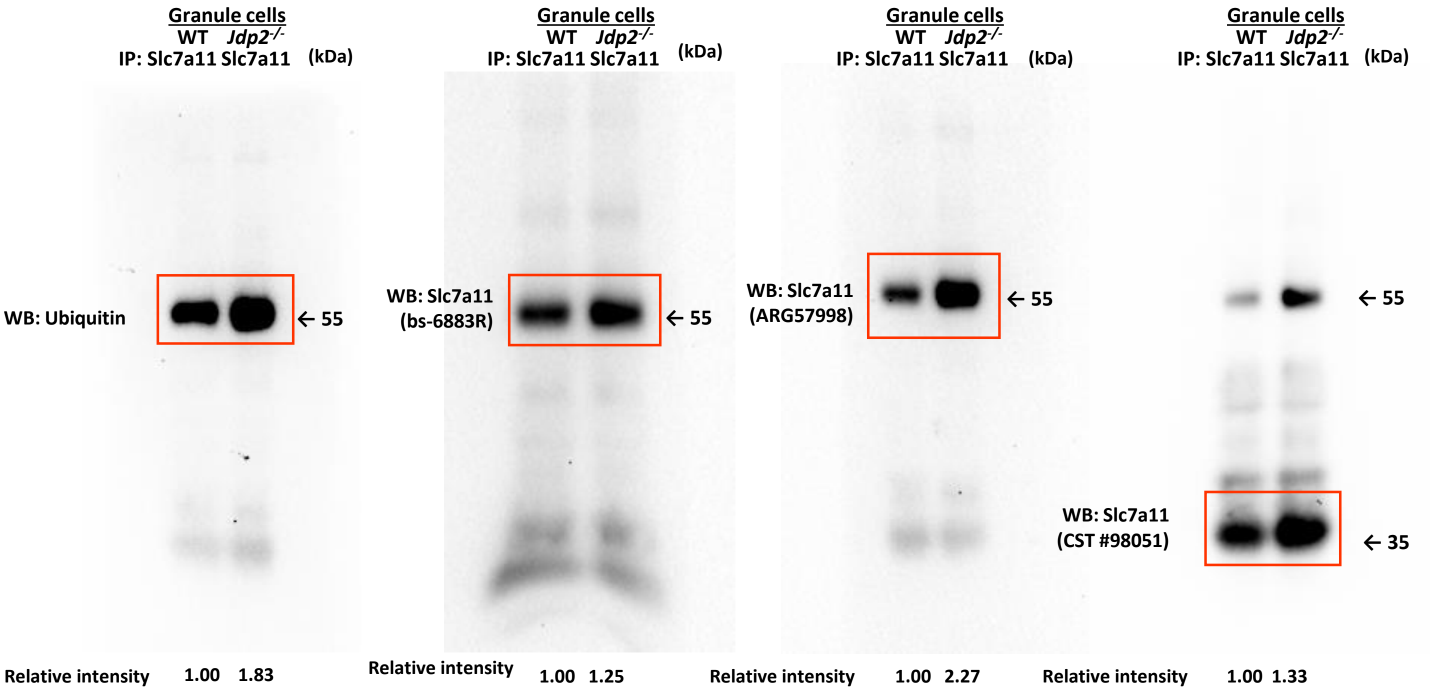

Supplementary Figure 7

STable 1 Antibodies used in this study

Antibody name	Company	Cat. no.	RRIDs
Nfm	Abcam	ab7794	RRID:AB_306083
Atoh1	Merck Millipore	AB5692	RRID:AB_91981
Gfp	Cell Signaling Technology	CST#2956	RRID:AB_1196615
p53	Cell Signaling Technology	CST#2524	RRID:AB_331743
$p21^{Cip1}$	Santa Cruz Biotechnology	sc-397	RRID:AB_632126
Caspase 3	Cell Signaling Technology	CST#9662	RRID:AB_331439
Cleaved Caspase 3	Cell Signaling Technology	CST#9664	RRID:AB_2070042
Cytochrome C	Cell Signaling Technology	CST#4272	RRID:AB_2090454
Bad	Santa Cruz Biotechnology	sc-7869	RRID:AB_634256
Bax	Cell Signaling Technology	CST#2772	RRID:AB_10695870
HIF-1β/Arnt	Cell Signaling Technology	CST#5537	RRID:AB_10694232
AhR	Santa Cruz Biotechnology	sc-8088	RRID:AB_2223957
Nrf2	Santa Cruz Biotechnology	sc-722	RRID:AB_2108502
Nrf2	Cell Signaling Technology	CST#14596	RRID:AB_2798531
Nrf2	Cell Signaling Technology	CST#12721	RRID:AB_2715528
MafF	MyBioSource	MBS835421	RRID:AB_2815026
MafG	MyBioSource	MBS9212444	RRID:AB_2815027
MafK	Abcam	ab50322	RRID:AB_881008
MafK (NF-E2p18)	Santa Cruz Biotechnology	sc-477	RRID:AB_2137821
Keap1	Cell Signaling Technology	GST#8047	RRID:AB_10860776
Jdp2	Abcam	ab40916	RRID:AB_943893
Jdp2	A gift from Dr. Aronheim		
Ho1	Santa Cruz Biotechnology	sc-10789	RRID:AB_648281
HDAC1	Cell Signaling Technology	CST#5356	RRID:AB_10612242
HDAC2	Cell Signaling Technology	CST#5113	RRID:AB_10624871
HDAC3	Cell Signaling Technology	CST#2632	RRID:AB_331545
HDAC3	Cell Signaling Technology	CST#85057	RRID:AB_2800047
Nqo1	Santa Cruz Biotechnology	sc-16464	RRID:AB_2154339
P300	Abcam	ab10485	RRID:AB_297224
P300	Abcam	ab3164	RRID:AB_303567
CBP	Santa Cruz Biotechnology	sc-369X	RRID:AB_631006
CBP	Cell Signaling Technology	CST#7389	RRID:AB_2616020
Cd44 (9A4)	Thermo Fisher Scientific	BMS145	RRID:AB_10597738
Slc7a11 (CCBR1)	Bioss Inc.	bs-6883R	RRID:AB_2815028
Slc7a11	Novus Biologicals	NB300-318	RRID:AB_527560

Slc7a11	Cell Signaling Technology	CST#98051	RRID:AB_2800296
Slc7a11	Arigo Biolaboratories	ARG57998	RRID:AB_2815029
β-actin	Santa Cruz Biotechnology	sc-47778	RRID:AB_2714189
Ubiquitin	Cell Signaling Technology	CST#3933	RRID:AB_2180538
Normal Rabbit IgG	Cell Signaling Technology	CST#2729	RRID:AB_1031062
Anti-Rabbit-IgG HRP	Cell Signaling Technology	CST#7074	RRID:AB_2099233
Anti-Mouse-IgG HRP	Cell Signaling Technology	CST#7076	RRID:AB_330924
Anti-Goat-IgG HRP	Santa Cruz Biotechnology	sc-2020	RRID:AB_631728
Anti-mIgGk HRP	Santa Cruz Biotechnology	sc-516102	RRID:AB_2687626
Anti-Goat IgG HRP	Jackson ImmunoResearch Inc.	# 705-035-147	RRID: AB_2313587
Anti-Rat-IgG HRP	Jackson ImmunoResearch Inc.	# 112-035-167	RRID: AB_2338139
Alexa-Fluor® 488 conjugate Goat anti-Mouse IgG	Thermo Fisher Scientific	A-11029	RRID:AB_138404
Alexa Fluor® 488 conjugate Goat anti-rabbit IgG	Thermo Fisher Scientific	A-11034	RRID:AB_2576217
Alexa Fluor® 594 conjugate Goat anti-mouse IgG	Thermo Fisher Scientific	A11032	RRID:AB_2534091
Alexa-Fluor® 594 conjugate Goat anti-Rabbit IgG	Thermo Fisher Scientific	A-11037	RRID:AB_2534095
Alexa Fluor® 647 Conjugate Goat anti-rat IgG	Cell Signaling Technology	CST#4418	RRID:AB_1904017

STable 2 siRNA used in this study

**Antibody name**	**Santa Cruz. cat. no.**	**Other company's**
Control siRNA (m)	Sc-44234	Santa Cruz Biotechnology
p21^{Cip1} siRNA (m)		Ambion® Thermo Fisher Scientific<br>Cat#4390771; siRNA ID #s63812 (#No.1)<br>Sigma-Aldrich<br>NM_00111-97 (#No.2)<br>NM_007669 (#No.3)
Slc7a11 siRNA (m)		Ambion® Thermo Fisher Scientific<br>Cat#4390771; siRNA ID #s77275 (#No.1); siRNA ID #s77276 (#No.2); siRNA ID #s77277 (#No.3)
Negative Control #01 siRNA		Ambion® Thermo Fisher Scientific<br>Cat#4390844

STable 3 Primers for qPCR used in this study

qPCR primers	Primer sequences (5’-3’)	
mGAPDH	Sense	ACCACAGTCCATGCCATCAC
	Antisense	TCCACCACCCTGTTGCTGTA
mp53	Sense	AAAGAGAGCGCTGCCCACCT
	Antisense	CTCCCGGAACATCTCGAAGC
mp21^{Cip}	Sense	ACGTGGCCTTGTCGCTGTCT
	Antisense	GACCAATCTGCGCTTGGAGTG
mAtoh1	Sense	GCTGTGCAAGCTGAAGGG
	Antisense	TCTTGTTGTTGAAGG
mHo1	Sense	GACAGAAGAGGCTAAGACCGC
	Antisense	TGGAGGAGCGGTGTCTGG
mNqo1	Sense	AGCTGGAAGCTGCAGACCTG
	Antisense	CCTTTCAGAATGGCTGGCA
mCD44va	Sense	AGCAGCGGCTCCACCATCGAG
	Antisense	TCGGATCCATGAGTCACAGTG
mSlc7a11	Sense	TGCAATCAAGCTCSTGAC
	Antisense	AGCTGTATAACTCCAGGGACTA
mAtoh1	Sense	ATGCACGGGCTGAACCA
	Antisense	TCGTTGTTGAAGGACGGGATA
mGclc	Sense	AACTAGCGAAGAGTTGGGCTTGGA
	Antisense	AAGTTAACGGGTCGCGTATCCACA
mGclm	Sense	AGCCAATCTGGAAGGAGATGCAGT
	Antisense	TTCTGCAGGGTCGTTATGGGTCAA
mGls1	Sense	GCACTACACTTTGGACACCA
	Antisense	TAGCAACCCGTCGAGATT
mGls2	Sense	ACCATGTGGTTGCTGGGATT
	Antisense	GATGCCCTCTTCTGGTGTGT
mASCT2	Sense	CCGCTGATGATGAAGTGC
	Antisense	CCCCCGATAGTGTTTGAG
mGad1	Sense	AGATAGCCCTGAGCGACGAG
	Antisense	ATGGCCGATGACCATGC
mGSR	Sense	ACTTGCGTGAATGTTGGATGTG
	Antisense	GCCGTAATCCACGTGATCGT
mPGD	Sense	CGGATCATCCTCCTGGTG
	Antisense	ATGATGTCACCAGTATCCAACAA
mHK2	Sense	GCCAGCCTCTCCTGATTTAGTGT

	Antisense	GGGAACACAAAAGACCTCTTCTGG
mSnnt1	Sense	TTGCTGTGTATACTGCCCTGGT
	Antisense	TCTCCTCCAGCTGGTCATAGTTGA
mShmt2	Sense	CCCTATGTTCCGCGAGTACTCCTT
	Antisense	TGTCAGTGCCACCAGACACCA
mPhgdh	Sense	TGTCTTCAGACCAGAGGTGCCACTA
	Antisense	ACACCATGGAGGTTTGGTAGGACA
mZic1	Sense	ATGAAGGTCCACGAAGCATC
	Antisense	CGTGCTGTGATTGGACGTGT
mZic2	Sense	GTCCACGCCTCCGATAGCC
	Antisense	CTCATGGACCTTCATGTGCT
mNGF	Sense	ACACTCTGATCACTGCGTTTTTG
	Antisense	CCTTCTGGGACATTGCTATATCTGT
mBDNF	Sense	CACTGGCTGACACTTTTGAGCAC
	Antisense	GCTGTGACCCACTCGCTAATACTG
mN-Myc	Sense	CTGAGCTGGTGAAGAACGAGAA
	Antisense	CTCGGTGGCCTTTTTCAAGA
mNdrg2	Sense	GAGTTAGCTGCCCGCATCC
	Antisense	GTGACCGAGCCATAAGGTGTC
mNdrg3	Sense	TCCCCAGCGGGTATCGTT
	Antisense	CATCACGGAGGGCAGCAT
mGabra1	Sense	AAAAGCGTGGTTCCAGAAAA
	Antisense	GCTGGTTGCTGTAGGAGCAT
mGabra2	Sense	GCTACGCTTACACAACCTCAGA
	Antisense	GACTGGCCCAGCAAATCATACT
mGlra2	Sense	TATTGCACAAAGCATTACAACA
	Antisense	CATTTTACCTTCCTATTTTATTTTG
mNTrk2	Origene Tech. MP208864	
mNTrk3	Origene Tech. MP208865	
mNT3	Origene Tech. MP208866	
mNeuroD1	Origene Tech. MP208806	
mGAPDH	Origene Tech. MP205604	
